# CD8^+^ tissue-resident memory T-cell development depends on infection-matching regulatory T-cell types

**DOI:** 10.1038/s41467-023-41364-w

**Published:** 2023-09-11

**Authors:** Leandro Barros, Daryna Piontkivska, Patrícia Figueiredo-Campos, Júlia Fanczal, Sofia Pereira Ribeiro, Marta Baptista, Silvia Ariotti, Nuno Santos, Maria João Amorim, Cristina Silva Pereira, Marc Veldhoen, Cristina Ferreira

**Affiliations:** 1 Instituto de Medicina Molecular | João Lobo Antunes, Faculdade de Medicina da Universidade de Lisboa, Av. Professor Egas Moniz, Lisbon, 1649-028 Portugal; 2 Instituto de Tecnologia Química e Biológica António Xavier, Av. da República, Oeiras, 2780-157 Portugal; 3 Instituto Gulbenkian de Ciência, Rua da Quinta Grande 6, Oeiras, 2780-156 Portugal; 4 Universidade Católica Portuguesa, Católica Médical School, Católica Biomedical Research Centre, Palma de Cima, 1649-023 Portugal; 5 Present Address: The Francis Crick Institute, 1 Midland Road, London, NW1 1AT UK

**Keywords:** Fungal infection, Viral infection, Parasitic infection, Cytotoxic T cells

## Abstract

Immunological memory is critical for immune protection, particularly at epithelial sites, which are under constant risk of pathogen invasions. To counter invading pathogens, CD8^+^ memory T cells develop at the location of infection: tissue-resident memory T cells (T_RM_). CD8^+^ T-cell responses are associated with type-1 infections and type-1 regulatory T cells (T_REG_) are important for CD8^+^ T-cell development, however, if CD8^+^ T_RM_ cells develop under other infection types and require immune type-specific T_REG_ cells is unknown. We used three distinct lung infection models, to show that type-2 helminth infection does not establish CD8^+^ T_RM_ cells. Intracellular (type-1) and extracellular (type-3) infections do and rely on the recruitment of response type-matching T_REG_ population contributing transforming growth factor-β. Nevertheless, type-1 T_REG_ cells remain the most important population for T_RM_ cell development. Once established, T_RM_ cells maintain their immune type profile. These results may have implications in the development of vaccines inducing CD8^+^ T_RM_ cells.

## Abstract (web summary)

Type-1 regulatory T cells promoted the generation of CD8+ tissue-resident memory T (TRM) cells during intracellular infections in the gut. Here, the authors show that the establishment TRM cells more broadly depends on the presence of regulatory T cells matching the type of infection.

## Introduction

Epithelial barriers are prime portals for microbial invasion. Organ structure, such as a multilayer of epithelial cells in the skin, provides additional protection against invasion. Other organs, such as the intestine and lung require optimal exchange of nutrients, liquids, and gasses that necessitate a single epithelial cell layer. It is highly beneficial to the host to contain invading pathogens at the site of entry and swiftly clear any pathogens and their products to avoid tissue damage and systemic dissemination of infectious or toxic material. For this reason, the immune response is tailored to the identity of the invading microbe, towards intracellular (type-1), helminths (type-2), or extracellular (type-3) pathogens. Understanding how tissue immunity is established following distinct responses has conceivable therapeutic potential with the ability to modulate tissue-specific immunity, from vaccination strategies to tumour treatment.

The development of immunological memory is a pillar of adaptive immunity and an efficacious way to prevent the development of disease caused by pathogen entry. Memory T lymphocytes are functionally and phenotypically classified in at least three subsets. Central memory (T_CM_) and effector memory (T_EM_) T cells recirculate throughout lymphoid and non-lymphoid organs respectively^1^. T_EM_ cells actively scout for pathogens, entering and exiting organs via the circulation and lymph, while T_CM_ cells are more quiescent and are reactivated in secondary lymphoid organs, faster than, but not dissimilarly from, naïve T cells^2,3^. Although these cells provide significant protection from recurrent infections due to increased antigen-specific numbers and speed of activation, they require a systemic immune reaction for triggering and may not be sufficiently efficacious against pathogen entry. Tissue-resident memory (T_RM_) T cells reside in tissues, especially those at the host/environment interface. T_RM_ cells actively survey the tissues for signs of infection, able to react rapidly and thereby swiftly eliminate infected cells without the need for a systemic immune reaction^1^.

CD8^+^ T_RM_ cells are characterised by the expression of different markers and transcription factors, which depend on their activation status and on the tissue type they home to^4^. Most T_RM_ cells constitutively express CD69, which antagonises the egress receptor S1P receptor 1, thus maintaining tissue residency^5–7^, with organ specificity^8^. The integrins CD103 and CD49a are additional markers of tissue residency, although T_RM_ cells negative for CD103 have been reported and CD49a may reflect activation status^9–12^. CD103, integrin αE pairing with β7, is mostly present on epithelial residing T_RM_ cells, providing support for tissue homing and maintenance via epithelial cell E-cadherin binding, while potentiating cytolytic activity. Of additional importance is the absence of Killer Cell Lectin Like Receptor G1 (KLRG1), typically described as a terminal differentiation marker on effector T cells^13,14^. KLRG1 may compete with CD103 for the binding to E-cadherin, with its downregulation contributing to T_RM_ cell residency^15^.

A transcriptional programme, distinct from other CD8^+^ T-cell subsets, drives surface markers and function of T_RM_ cells. T_RM_ cells express low levels of Tbox protein expressed in T cells (Tbet) and Krüppel-like Factor 2 (KLF2), while Eomesodermin (Eomes) is absent^6,14,16^. Instead, T_RM_ cells express high levels of arylhydrocarbon receptor (AhR) in gut and skin, Hobit and Blimp-1^17,18^. In addition, T_RM_ cells are maintained in a semi-activation state and distinct metabolic wiring takes place, ensuring rapid full activation when required^19,20^.Thus, T_RM_ cells patrol non-lymphoid organs and perform immunosurveillance by killing infected cells and producing cytokines to recruit other immune cells to quickly eliminate the pathogen^21,22^.

Exacerbated T-cell responses can result in tissue damage, and a balance between pathogen elimination and immunopathology needs to be maintained. Regulatory T cells (T_REG_), characterised by the expression of Forkhead box protein 3 (Foxp3) transcription factor, play an important role in reducing excessive immune responses. T_REG_ can influence the transition of effector CD8^+^ T cells to memory cells by limiting interleukin (Il)−2 availability, producing Il-10 and cytotoxic T-lymphocyte associate protein-4 (CTLA-4)^23–25^. Of importance, T_REG_ cells can adopt a transcriptional programme in line with the type of inflammation and the CD4^+^ T helper (T_H_) subset involved. Besides Foxp3, T_REG_ cells can co-express the T_H_1 cell (type-1) factor Tbet, the T_H_2 cell (type-2) factor Gata-3, and the T_H_17 cell (type-3) factor Rorγt, to take on characteristics of one of these T_H_ cell ineages^22,26–28^.

We previously showed that type-1 T_REG_ cells, recruited via their Tbet-dependent expression of the chemokine receptor CXCR3, supply and activate transforming growth factor (TGF)β locally, thereby efficiently facilitating the generation of T_RM_ cells^14^. However, although T_RM_ cells were found reduced in several organs, the link between their generation and type-1 T_REG_ cells was established in the intestine and during a type-1 immune response. This raises the question of whether the establishment of T_RM_ cells in another organ is also dependent on type-1 T_REG_ cells, and more importantly, whether T_RM_ cell development during other types of infection is dependent on the corresponding type of T_REG_ cell or remains dependent on type-1 T_REG_ cells. Here, we show that, in the lung, type-1 T_REG_ cells are required to establish T_RM_ cells upon type-1 infection, while a type-2 infection does not establish a robust T_RM_ cell population. Furthermore, type-3 infection in the same organ requires type-3 T_REG_ cells and their provision of TGFβ for efficient T_RM_ cell establishment.

## Results

### Type-1 immunity induces T_RM_ cells in lungs

**Fig. 1:**
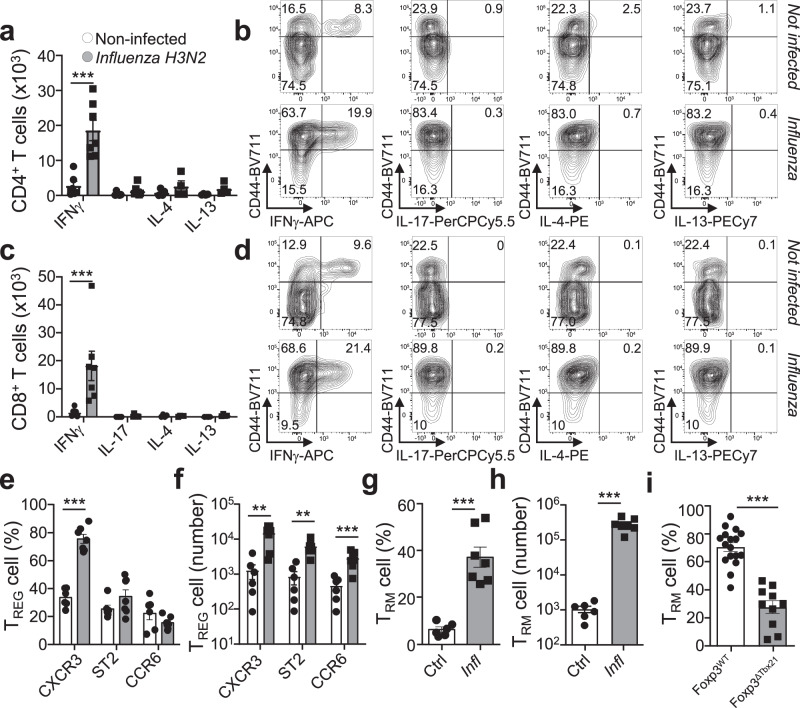
Type-1 TREG cells are required for TRM cell generation in the lungs upon Influenza infection. Mice were infected or not intranasally with 1000 plaque-forming units (PFU) of Influenza A X-31 strain (H3N2). 10 days post-infection lungs were collected, and cells were isolated and analysed via flow cytometry for cytokine production. **a** Numbers of activated (CD44^+^) CD4^+^ T cells producing indicated cytokines (*p*_(Non-infected vs. Influenza H3N2)_ < 0.000001) and (**b**) representative flow cytometry plots (*n* = 6, non-infected, *n* = 7 infected, *N* = 3). **c** Numbers of activated CD8^+^ T cells producing indicated cytokines (*p*_(Non-infected vs. Influenza H3N2)_ = 0.000013) and **d** representative flow cytometry plots (*n* = 6, non-infected, *n* = 7 infected, *N* = 3). **e**, **f** Foxp3^WT^ mice (*n* = 6, non-infected, *n* = 7 infected, *N* = 3) were assessed for **e** percentage (*p*_(Non-infected vs. Influenza H3N2)_ <0.000001), and **f** numbers of chemokine receptors CXCR3, ST2 (IL-33R) and CCR6 expression (*p*_(Non-infected vs Influenza H3N2)_: CXCR3, *p* = 001415; ST2, *p* = 0.000502; CCR6, *p* = 0.001132), on T_REG_ cells (*n* = 6, non-infected, *n* = 8 infected, *N* = 3). **g**, **h** Mice analysed via flow cytometry for **g** percentages of total CD8^+^ T cells and **h** numbers of T_RM_ cells, defined as CD69^+^KLRG1^−^ CD8^+^ T cells, *p*(_Non_^-^_infected vs Influenza H3N2)_ < 0.0001 (*n* = 6, *N* = 3). **i** Foxp3^WT^ and Foxp3^ΔTbx21^ mice received CD8^CD45.1^ T cells intravenously, one day prior to infection. 14 days post infection, lung cells were assessed by flow cytometry for the percentage of T_RM_ cells (CD69^+^KLRG1^-^CD103+) in the transferred population (Foxp3^WT^ *n* = 17, Foxp3^ΔTbx21^ *n* = 10, *N* = 4), *p*_(Foxp3WT vs Foxp3ΔTbx21)_ <0.0001. Two-sided Mann–Whitney analysis was applied to compare groups. Data are presented as bars of mean ± SEM with single data points.

In the absence of type-1 T_REG_, there is a reduction in CD8^+^ T_RM_ cells in the intestine, lungs, and liver, due to inhibition of their generation^14^. To establish a robust infection model in the lungs, we infected Foxp3^WT^ mice with influenza H3N2 (X-31). A week after influenza infection, a robust influx of CD4^+^ and CD8^+^ T cells is observed (Supplementary Fig. 1a–d). Influenza challenge results in a strongly polarised type-1 response with production of interferon (IFN)-γ and dominance of CXCR3-expressing CD4^+^ and CD8^+^ T cells, with no other polarised T-cell response observed (Fig. 1a–d; Supplementary Fig. 1e–h). In addition to the type-1 response of CD4^+^ and CD8^+^ T cells, predominantly type-1 T_REG_ cells expressing CXCR3 (Fig. 1e–f) are recruited to the site of inflammation.

Influenza infection results in the establishment of a substantial CD8^+^ T_RM_ cell population (Fig. 1g–h). To understand if their establishment in the lungs upon influenza infection is dependent on type-1 T_REG_ cells we make use of our previously setup transfer model whereby CD45.1 expressing CD8^+^ T cells (CD8^CD45.1^) are transferred into control Foxp3^Wt^ mice or Foxp3^ΔTbx21^ mice that miss type-1 T_REG_^14^. Cells are readily recovered in the spleens, and the characteristic weight loss is observed (Supplementary Fig. 1i–k). As previously shown in the small intestine, the absence of type-1 T_REG_ cells reduces the establishment of CD8^+^ T_RM_ cells, after CD8^CD45.1^ transfer. We now extend this observation to the lungs upon influenza infection where compared to controls (Foxp3^WT^), the absence of type-1 T_REG_ cells leads to reduced efficiency of CD8^+^ T_RM_ cell development (Fig. 1i).

### Type-2 immunity does not induce de novo CD8^+^ T_RM_ cells in lungs

**Fig. 2:**
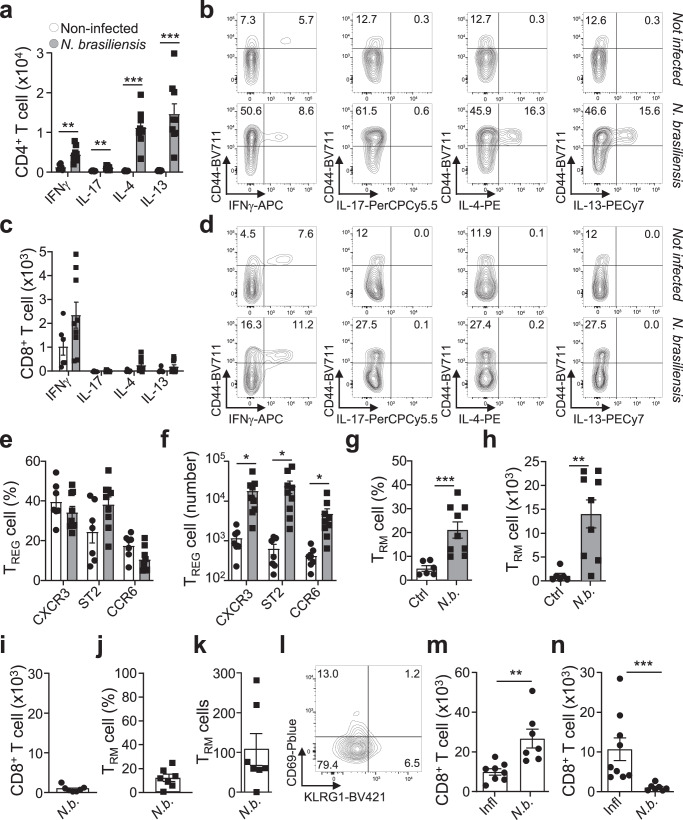
Type-2 Nippostrongylus brasiliensis infection does not yield CD8 TRM cell formation. Mice were subcutaneously infected or not with 300 stage L3 larvae of *Nippostrongylus brasiliensis*. 7 days post-infection lungs were collected, and cells were isolated and analysed via flow cytometry for cytokine production, and T_RM_ cells. **a** Numbers of activated (CD44^+^) CD4^+^ T cells producing indicated cytokines, *p*_(Non-infected vs *Nippostrongylus brasiliensis*)_: IFN-γ, *p* = 0.001749; IL-17, *p* = 0.002537; IL-4, *p* = 0.000048; IL-13, *p* = 0.000729, and **b** representative flow cytometry plots (non-infected *n* = 6, infected *n* = 9, *N* = 3). **c** Numbers of activated CD8^+^ T cells producing indicated cytokines and **d** Representative flow cytometry plots of cytokine production in CD8^+^ T cells (non-infected *n* = 6, infected *n* = 9, *N* = 3). **e**, **f** Foxp3^WT^ mice were assessed for **e** percentage and **f** numbers of chemokine receptors CXCR3, ST2 (IL-33R) and CCR6 expression on T_REG_ cells, *p*_(Non-infected vs *Nippostrongylus brasiliensis*)_: CXCR3, *p* = 0.014118; ST2, *p* = 0.023778; CCR6, *p* = 0.028885, (non-infected *n* = 6, infected *n* = 9, *N* = 3). **g**, **h** CD69^+^KLRG1^−^ CD8^+^ T_RM_ cell **g** percentage of total CD8^+^ T cells, *p*_(Non-infected vs *Nippostrongylus brasiliensis*)_ = 0.0004, and **h** number, *p*_(Non-infected vs *Nippostrongylus brasiliensis*)_= = 0.0016, in non-infected (control) and infected mice (non-infected *n* = 6, infected *n* = 9, *N* = 3). **i**–**l** Foxp3^WT^ mice received CD8^CD45.1^ T cells intravenously, one day prior to infection. 14 days post-infection, lung cells were analysed via flow cytometry for **i** total number of CD8^CD45.1^ T cells, CD69^+^KLRG1^−^ CD8^+^ T_RM_ cell **j** percentage and **k** numbers, and **l** representative flow plot, within the CD8^CD45.1^ T-cell population (*n* = 7, *N* = 3). **m**, **n** Comparison of transferred CD8^CD45.1^ T-cell number in the (**m**) spleen, *p*_(Influenza vs *Nippostrongylus brasiliensis*)_ = 0.0012, or (**n**) lungs, p_(Influenza vs *Nippostrongylus brasiliensis*)_ = 0.0002, of mice infected with *Influenza* and *N. brasiliensis* (*Influenza* *n* = 9, *N. brasiliensis* *n* = 7, *N* = 3). 2-sided Mann–Whitney analysis was applied to compare the differences between groups. Data is presented as bars of mean ± SEM with single data points.

Establishment of CD8^+^ T_RM_ cells in the lung with similar requirements to the small intestine may still rely on the type-1 inflammation induced by an intracellular pathogen as influenza virus. We subsequently used the helminth *Nippostrongylus brasiliensis*, widely used as a model to elicit strong type-2 immunity in the lungs^29^. A week after infection we observe a strong activation of CD4^+^ T cells, CD44^hi^, and a response characterised by T_H_2 cells in the lungs expressing IL-4 and IL-13 (Fig. 2a, b; Supplementary Fig. 2a, b)^30^. However, the CD8^+^ T-cell compartment shows a very limited effector CD44^hi^ CD8^+^ T-cell response, without type-2 polarisation, with primarily IFN-γ produced (Fig. 2c, d). There is an influx of T_REG_ cells, but without a polarisation towards any specific T_REG_ subset (Fig. 2e, f). Although some endogenous CD8^+^ T_RM_ cell accumulation is observed, the CD8 T-cell activation in the lungs is modest (Fig. 2g, h, Supplementary Fig. 2c, d).

CD8^CD45.1^ and CD4^CD45.1^ T cells transferred into Foxp3^Wt^ mice are recovered from the spleen, with robust numbers but without signs of activation with the majority of cells showing a naïve or T_CM_ cell phenotype (Supplementary Fig. 2e–g). CD8^CD45.1^ T cells are few in the lungs upon infection, with CD8^+^ T_RM_ cells rare and often below the detection threshold (Fig. 2i–l). CD4^CD45.1^ T cells are more numerous in the lungs upon infection, at cost of cell numbers in the spleen, with CD4^+^ T_RM_ cell proportions much higher than CD8^+^ T_RM_ cells (Supplementary Fig. 2g–h). This indicated robust CD4^+^ T-cell recruitment to the site of *N*. *brasiliensis* infection as opposed to CD8^+^ T cells. Comparing influenza and *N*. *brasiliensis*, CD8^CD45.1^ T cells remain abundant upon type-2 infection in the spleen, while transferred CD8^+^ T cells are recruited to the site of infection only after challenge with influenza (Fig. 2m, n). Although CD8^+^ T_RM_ cell generation is efficient in lung tissue, with CD8^+^ T cells recruited and relying on type-1 T_REG_ cell help during a type-1 immune response, a type-2 infection, despite a robust CD4^+^ T-cell response (Fig. 2a, b), does not result in de novo CD8^+^ T-cell recruitment, activation or polarisation, the recruitment of type-2 T_REG_ cells, or the establishment of CD8^+^ T_RM_ cells.

### Type-3 immunity does induce CD8^+^ T_RM_ cells in lungs

**Fig. 3:**
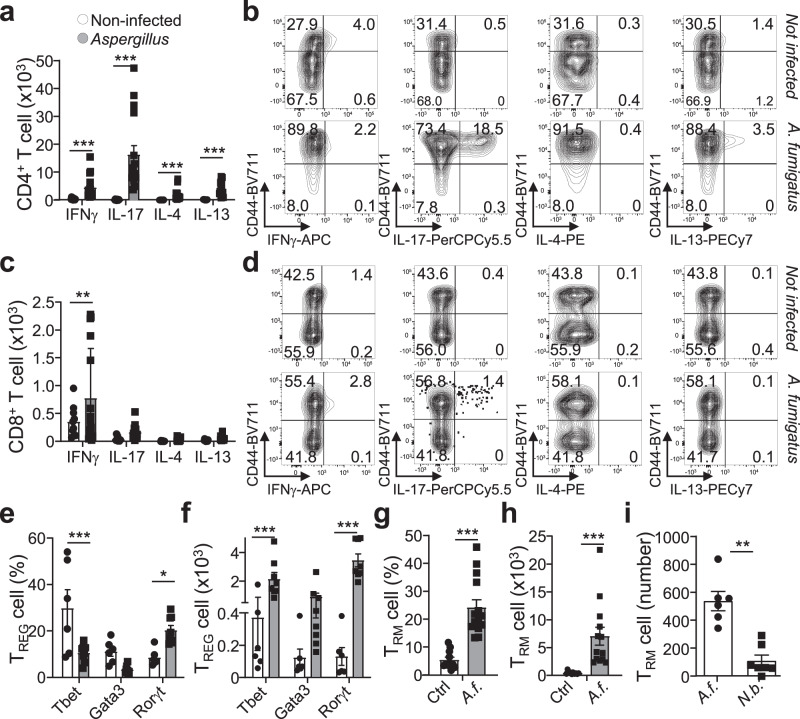
Type-3 Aspergillus fumigatus infection yields CD8 T-cell recruitment and TRM cell formation. Mice were intranasally challenged four times every 3 days with 10^6^ spores of *Aspergillus fumigatus*. 10 days post-infection lungs were collected, and cells were isolated and analysed via flow cytometry for cytokine production, and T_RM_ cells. **a** Numbers of activated (CD44^+^) CD4^+^ T cells producing indicated cytokines, *p*_(Non-infected vs *Aspergillus*)_: IFN-γ, *p* = 0.005452; IL-17, *p* = 0.000733; IL-4, *p* = 0.007702; IL-13, *p* = 0.000005, and **b** representative flow cytometry plots of cytokine production in CD4^+^ T cells (non-infected *n* = 10, infected *n* = 12, *N* = 4). **c** Numbers of activated CD8^+^ T cells producing indicated cytokines, *p*_(Non-infected vs *Aspergillus*)_: IFN-γ, *p* = 0.000951, and **d** representative flow cytometry plots of cytokine production in CD8^+^ T cells (non-infected *n* = 10, infected *n* = 14, *N* = 4). **e**, **f** T_REG_ cells from Foxp3^WT^ mice were assessed for expression in **e** percentage, *p*_(Non-infected vs *Aspergillus*)_: Tbet, *p* = 0.018008; RORγt, *p* = 0.001023, and **f** numbers, *p*_(Non-infected vs *Aspergillus*)_: Tbet, *p* = 0.022810; RORγt, *p* = 0.000093, of the transcription factors Tbet, GATA-3 and RORγt in (non-infected *n* = 6, infected *n* = 7, *N* = 3). **g**, **h** Analysis of CD69^+^KLRG1^-^ CD8^+^ T_RM_ cell (**g**) percentage, *p*_(Non-infected vs *Aspergillus*)_ = 0.000017, within the total CD8^+^ T-cell population, and **h** number, *p*_(Non-infected vs *Aspergillus*)_ = 0.002175 in the lungs of non-infected (control) and infected mice (*n* = 10, non-infected, *n* = 14 infected, *N* = 3). **i** Comparison of transferred CD8^CD45.1^ CD69^+^KLRG1^−^ CD8^+^ T_RM_ cell numbers in the lungs upon *Nippostrongylus brasiliensis* (N.b.) or *Aspergillus fumigatus* (A.f.) infection, *p*_(Aspergillus vs Nippostrongylus brasiliensis)_=0.0012, (*n* = 7, *N* = 3 for N.b.; *n* = 6 and *N* = 3 for A.f). Two-sided Mann-Whitney analysis was applied to compare the differences between groups. Data is presented as bars of mean ± SEM with single data points.

Besides intracellular pathogens resulting in robust CD8^+^ T_RM_ cell development, we asked if extracellular pathogens, not helminths, would result in the generation of CD8^+^ T_RM_ cells. *Aspergillus fumigatus* infection results in a robust response dominated by T_H_17 cells (Fig. 3a, b; Supplementary Fig. 3a, b)^31^. In contrast to a type-2 infection, a type-3 challenge shows a type-1 response with increased numbers of T_H_1 accompanied by a robust CD44^hi^ effector CD8^+^ T-cell response, but with few expressing IFN-γ, yet IL-17 production is observed (Fig. 3c, d; Supplementary Fig. 3c, d).

The influx of T_REG_ cells shows skewing towards type-3 T_REG_ at cost of type-1 T_REG_ cells, with total T_REG_ cell numbers increasing for all subsets (Fig. 3e, f). The T_REG_ cell response reflects the CD4^+^ T-cell response, which shows strong skewing towards Rorγt^+^CCR6^+^ T_H_17 cells, but with increased numbers in all subsets (Supplementary Fig. 3e–g). The proportional distribution of CD8^+^ T cells remains similar to uninfected, but with total numbers of type-3 CD8^+^ T cells increasing (Supplementary Fig. 3h–j). *Aspergillus* infection increases endogenous host CD8^+^ T_RM_ cell numbers in the lungs (Fig. 3g, h). Upon adoptive transfer of CD8^CD45.1^ T cells, in contrast to the type-2 infection used, newly established CD8^+^ T_RM_ cells were more robustly detected upon *Aspergillus* challenge compared to *N*. *brasiliensis* infection (Fig. 3i). In summary, type-1 and 3 infections result in CD8^+^ T_RM_ cell formation, which is not observed for a type-2 immune response.

### Deletion of Rorγt in FoxP3^+^ cells ablates type-3 T_REG_ cells

**Fig. 4:**
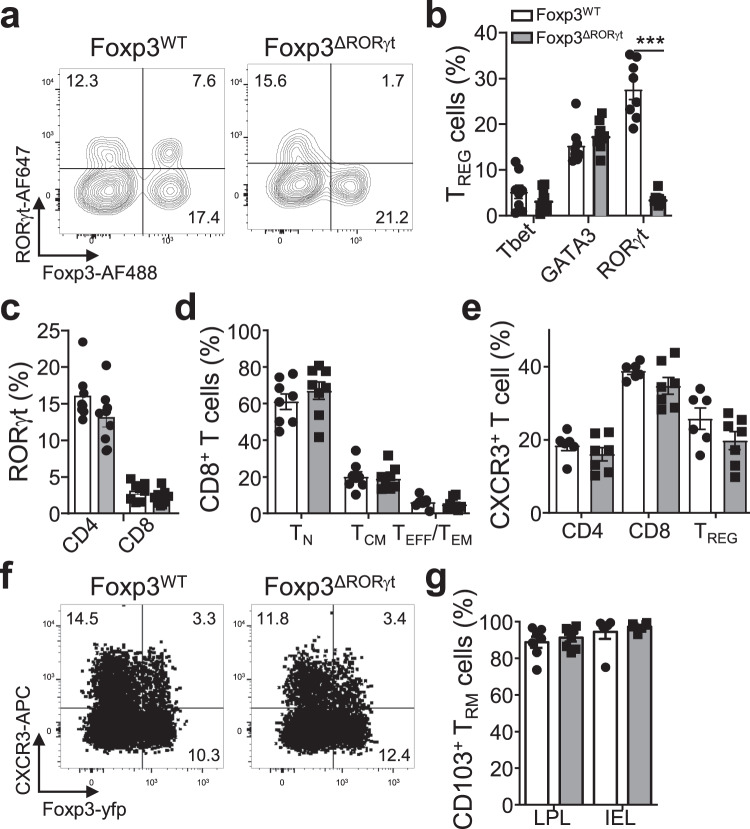
Type-3 TREG cells are ablated in Foxp3-CreeYFP RORγtfl/fl mice. Foxp3^WT^ and Foxp3^ΔRORγt^ mice were analysed at steady-state for T_REG_, CD4^+^, and CD8^+^ T cells in lung, spleen, small intestine lamina propria lymphocytes (LPL), and small intestine intraepithelial lymphocytes (IEL). **a** Representative flow plot of RORγt expression in LPL T_REG_ cells in both mouse lines. **b** Percentage of LPL T_REG_ cells expressing Tbet, GATA-3 or RORγt, p_(Foxp3WT vs Foxp3ΔRORγt)_ < 0.000001, (Foxp3^WT^ *n* = 8, Foxp3^ΔRORγt^ *n* = 9, *N* = 3). **c** Percentage of RORγt expression in CD4^+^ and CD8^+^ LPL (Foxp3^WT^ *n* = 8, Foxp3^ΔRORγt^ *n* = 9, *N* = 3). **d**–**f** CD8^+^ T-cell proportions in the spleen were assessed for **d** naive (T_N_ CD44^−^CD62L^+^), central memory (T_CM_, CD44^+^CD62L^+^) and effector memory/effector (T_EM_/T_EFF_, CD44^+^CD62L^−^) and **e** for CXCR3 expression on CD4^+^, CD8 and T_REG_ cells, with **f** representative flow plot (*n* = 8, *N* = 3). **g** Percentage of CD69^+^KLRG1^-^CD103^+^ CD8^+^ T_RM_ cell in small intestine LPL and IEL in both mouse lines (LPL Foxp3^WT^ *n* = 7, Foxp3^ΔRORγt^ *n* = 8, *N* = 3^;^ IELs *n* = 6, *N* = 2). Two-sided Mann–Whitney analysis was applied to compare the differences between groups. Data is presented as bars of mean ± SEM with single data points.

Our results using the type-3 immunity-inducing fungi *Aspergillus* indicate that CD8^+^ T_RM_ cells are induced during type-3 infections. This raises the question if T_REG_ cells are required and if the type of T_REG_ cell needs to match the infection type to efficiently establish CD8^+^ T_RM_ cells after type-3 infections. In order to address this, we generated Foxp3-yfp-Cre Rorγt^f/f^ mice (Foxp3^ΔRorγt^), in which type-3 T_REG_ cells are efficiently removed similarly as to type-1 T_REG_ cells in Foxp3^ΔTbx21^ mice, in all tissues assayed (Fig. 4a, b)^14^. CD4^+^ or CD8^+^ T-cell proportions or numbers, including Rorγt-expressing subsets such as T_H_17 cells, or T_REG_ subset proportions and numbers are not altered in Foxp3^ΔRorγt^ mice compared to controls (Fig. 4b–d; Supplementary Fig. 4a–d). Since type-1 T_REG_ cells are important in CD8^+^ T_RM_ cell formation^14^, we also determined proportion and numbers of type-1 T_REG_ cells and CXCR3 expression. These are similar between Foxp3^Wt^ and Foxp3^ΔRorγt^ mice (Fig. 4b, e, f; Supplementary Fig. 4c, e). In agreement with the presence of similar numbers of type-1 T_REG_ cells, the number of intraepithelial lymphocytes (IELs) and CD8^+^ T_RM_ cells in the lamina propria lymphocytes (LPL), lung, and liver, which differ in Foxp3^ΔTbx21^ mice^14^, were comparable between Foxp3^Wt^ and Foxp3^ΔRorγt^ mice (Fig. 4g; Supplementary Fig. 4f–h). Collectively, the data shows that efficient deletion of type-3 T_REG_ cells is established without effects on other T-cell populations, as well as a noticeable effect on CD8^+^ T_RM_ cells present at steady state.

### Deletion of type-3 T_REG_ cells impacts CD8^+^ T_RM_ cell formation

**Fig. 5:**
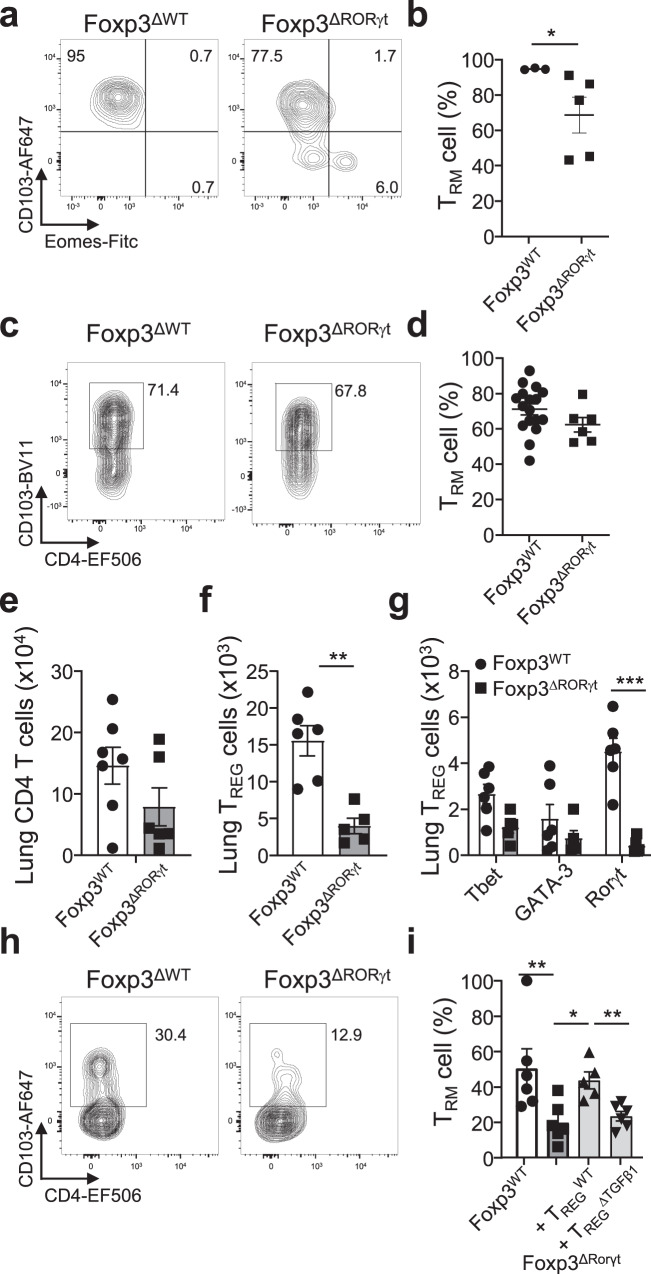
Depletion of Type-3 TREG cells reduces TRM cell development in type-3 infections. **a**, **b** In Foxp3^WT^ and Foxp3^ΔRORγt^ mice, CD45.1^+^ CD8^+^ T cells were transferred one day prior to infection with 100 mg of segmented filamentous bacteria (SFB)-containing feces, organs were assessed for their T_RM_ cell phenotype (CD69^+^Eomes^-^CD103^+^) 14 days later. Small intestine LPL were collected and assessed for the T-cell phenotype of CD45.1^+^ CD8^+^ T_RM_ cells. **a** Representative flow cytometry plots and **b** percentages of T_RM_ cells, *p*_(Foxp3WT vs Foxp3ΔRORγt)_ = 0.0357, (Foxp3^WT^ *n* = 3, Foxp3^ΔRORγt^ *n* = 5, *N* = 3). **c**, **d** Intranasal challenge with 1000 PFU of Influenza X-31 strain (H3N2). Lungs were collected and transferred T cells were analysed. **c** Representative flow cytometry plots of T_RM_ cells within transferred T cells and **d** percentages of T_RM_ cells in indicated mouse lines (Foxp3^WT^ *n* = 17, Foxp3^ΔRORγt^ *n* = 6, *N* = 4). **e**–**g** Mice were infected by four intranasal challenges with 10^6^ spores of *Aspergillus fumigatus*. Lungs were collected and **e** total CD4^+^ T cells (Foxp3^WT^ *n* = 7, Foxp3^ΔRORγt^ *n* = 6, *N* = 3), **f** total T_REG_ cells, *p*_(Foxp3WT vs Foxp3ΔRORγt)_=0.0043, and **g** subsets of T_REG_ cells based on their transcription factor expression, *p*_(Foxp3WT vs Foxp3ΔRORγt)_ = 0.000001, were analysed (Foxp3^WT^ *n* = 6 Foxp3^ΔRORγt^ *n* = 5, *N* = 3). **h**, **i** Mice were transferred with indicated condition receiving control or TGFβ1–deficient T_REG_ cells prior to the first of four intranasal challenges with 10^6^ spores of *Aspergillus fumigatus*. Lungs were collected and transferred CD45.1 CD8 T cells were analysed. **h** Representative flow cytometry plots of T_RM_ cells within transferred CD45.1 CD8 T cells and **i** percentages of T_RM_ cells in all conditions, *p*_(Foxp3WT vs Foxp3ΔRORγt)_ = 0.0087, *p*_(Foxp3ΔRORγt vs Foxp3ΔRORγt+WT Tregs)_ = 0.0173, *p*_(Foxp3ΔRORγt vs Foxp3ΔRORγt+TregΔTGFβ1)_ = 0.0043 (Foxp3^WT^; Foxp3^ΔRORγt^ *n* = 6, *N* = 4; Foxp3^ΔRORγt^ + T_REG_^WT^ *n* = 5, *N* = 2; Foxp3^ΔRORγt^ + T_REG_^ΔTGFβ1^ *n* = 5, *N* = 2). Multiple unpaired *t* test was used for **g**, other panels two-sided Mann-Whitney analysis was applied to compare groups. Data is presented as bars of mean ± SEM with single data points.

Segmented filamentous bacteria (SFB) are known inducers of T_H_17 cells^32^. However, in our hands, there was a modest induction of T_H_17 cells, accompanied by T_H_1 cells as well, resulting in a mixed type-1/type-3 response (Supplementary Fig. 5a, b). In accordance, T_REG_ cell recruitment was similar among the major three subsets (Supplementary Fig. 5c, d). Foxp3^ΔRorγt^ mice mount a similar T_H_1 and T_H_17 cell response compared to Foxp3^Wt^ controls (Supplementary Fig. 5e, f). SFB infection results in substantial CD8^+^ T_RM_ cell establishment, but with reduced development in the absence of type-3 T_REG_ cells (Fig. 5a, b). However, the chronicity of SFB in the intestinal tract prohibits the further assessment of memory T-cell responses^32^.

Influenza virus causes a strong type-1 response and establishment of CD8^+^ T_RM_ cells in the lung, dependent on type-1 T_REG_ cells (Fig. 1). Upon infection, Foxp3^ΔRorγt^ and Foxp3^Wt^ animals showed a similarly robust development of CD8^+^ T_RM_ cells (Fig. 5c, d), in stark contrast to Foxp3^ΔTbx21^ mice (Fig. 1i), indicating primarily a dependency on type-1 T_REG_ cells for CD8^+^ T_RM_ cell development. This raised the question if *Aspergillus* infection, resulting in a strong type-3 response (Fig. 3), requires type-3 T_REG_ cells for optimal CD8^+^ T_RM_ cell development. Upon *Aspergillus* infection the influx of CD4^+^ T cells is similar between Foxp3^Wt^ and Foxp3^ΔRorγt^ animals (Fig. 5e). However, the recruitment of T_REG_ cells is significantly lower (Fig. 5f), explained by the absence of type-3 T_REG_ cells (Fig. 5g). This is similar to our previous observations in type-1 infections in Foxp3^ΔTbx21^ animals^14^. Genetic ablation of type-3 T_REG_ cells in Foxp3^ΔRorγt^ animals resulted in a marked reduction of CD8^+^ T_RM_ development compared with Foxp3^Wt^ animals (Fig. 5g, h). Importantly, and in line with previous results obtained with type-1 pathogens (Fig. 1)^14^, co-transfer of control T_REG_ cells into type-3 T_REG_–deficient hosts prior to a type-3 infection is able to restore CD8^+^ T_RM_ cell development and numbers to the level seen in control mice, which also for type-3 responses relies on T_REG_ cell provision of TGFβ (Fig. 5i, Supplementary Fig. 5i).

Collectively, our data suggest that there appears to exist a matching of pathogen type and T_REG_ subset during type-1 and type-3 infections. Influenza, SFB and *Aspergillus*, show a dominant dependency of the equivalent T_REG_ subset for efficient CD8^+^ T_RM_ cell development.

### CD8^+^ T_RM_ cell identity is maintained after infection

**Fig. 6:**
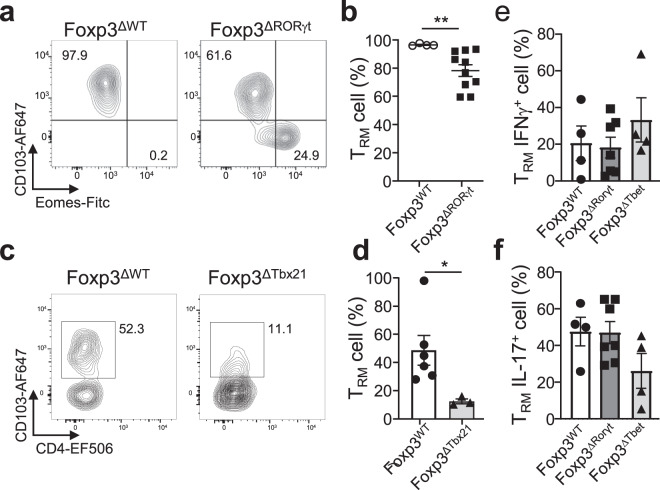
Type-1 and type-3 TREG cells enhance TRM cell development, the identity of which are maintained. Mice were transferred with CD45.1^+^ CD8^+^ T cells one day prior to oral gavage infection. 14 days after transplant, tissues were collected and assessed for their T_RM_ phenotype. **a**, **b** Infection with 5000 oocysts of *Eimeria vermiformis*, **a** representative flow cytometry plots T_RM_ cells (CD69^+^Eomes^-^CD103^+^) and **b** percentages of T_RM_ cells in both mouse lines, *p*_(Foxp3WT vs Foxp3ΔRORγt)_ = 0.0020, (Foxp3^WT^ *n* = 4, Foxp3^ΔRORγt^ *n* = 10, *N* = 3). **c**, **d** Mice were challenged four times intranasally with 10^6^ spores of *Aspergillus fumigatus*. Lungs were collected and transferred T cells were analysed, **c** representative flow cytometry plots of T_RM_ cells and **d** percentages of T_RM_ cells in both mouse lines, *p*_(Foxp3WT vs Foxp3ΔTbet)_ = 0.0238 (Foxp3^WT^ *n* = 6, *N* = 4, Foxp3^ΔTbx21^ *n* = 3, *N* = 3). **e**, **f** Mice were transferred with CD45.1 CD8 T cells prior to the first of four intranasal challenges with 10^6^ spores of *Aspergillus fumigatus*. Lungs were collected and transferred CD45.1 CD8^+^ T_RM_ cells were analysed for **e** IFNγ or **f** IL-17 production (Foxp3^WT^ *n* = 4, Foxp3^ΔRORγt^ *n* = 7, Foxp3^ΔTbx21^ *n* = 4, *N* = 3). Two-sided Mann–Whitney analysis was applied to compare groups. Data is presented as bars of mean ± SEM with single data points.

*Eimeria vermiformis* (Ev) is a small intestinal single-cell intracellular parasite that invades small intestine epithelial cells, it is mouse-specific and self-limiting, making it an ideal tool to study T_RM_ cell development^14^. It undergoes at least three rounds of asexual replication, each time bursting out of an infected epithelial cell to reach distal uninfected epithelial cells^33^. Its clearance depends on a type-1 inflammatory response, as we showed previously, and establishes type-1 T_REG_-dependent CD8^+^ T_RM_ cells^14^. We tested if the establishment of CD8^+^ T_RM_ cells is inhibited by the absence of type-3 T_REG_ cells in Foxp3^ΔRorγt^ mice. Foxp3^ΔRorγt^ mice do show reduced CD8^+^ T_RM_ cell development compared to Foxp3^Wt^ controls, although the decrease is not as marked as in the absence of type-1 T_REG_. (Fig. 6a, b)^14^.

CD8^+^ T cells are strongly associated with type-1 responses. We observed IFNγ-producing CD8^+^ T cells also in type-2 and type-3 infections (Figs. 2c, d, 3c, d). Hence, we next questioned if, in a strong type-3 infection model such as *Aspergillus*, the subsequent CD8^+^ T_RM_ cell establishment that is dependent on type-3 T_REG_ cells (Fig. 5e, f), shows any dependency on type-1 T_REG_ cells as is the case with Ev infection (Fig. 6a, b). In the case of *Aspergillus*, the absence of either type-3 or type-1 has marked effects on the generation of CD8^+^ T_RM_ cells (Figs. 5e, f, 6c, d).

Since memory T cells are maintained to offer protection against future challenges, we wished to know if CD8^+^ T_RM_ cells developed under polarising conditions of type-1 or type-3 infections reflected the original skewing. We assessed the endogenous CD8^+^ T_RM_ cell population after the clearance of infection. Upon influenza infection, the established CD8^+^ T_RM_ cells show a predominant skewing towards the original type-1 response, with the cells mainly able to produce IFNγ (Supplementary Fig. 6a–c). Upon an *Aspergillus* infection, the remaining CD8^+^ T_RM_ cells are fewer and the skewing is less apparent. The recall response shows IL-17 production, in line with the initial type-3 immune response, but there is the presence of IFNγ-producing CD8^+^ T_RM_ cells as well (Supplementary Fig. 6d–f). To analyse the polarisation of newly recruited CD8^+^ T cells upon an *Aspergillus* challenge at the site of infection, we made use of our CD45.1 T-cell adoptive transfer model. The *Aspergillus* challenge shows a mixed type-1 and type-3 polarisation of established CD8^+^ T_RM_ cells, indicating their polarisation is maintained after T_RM_ cell development (Fig. 6e–f). Furthermore, this model allows the assessment of the role of T_REG_ cell types on T_RM_ cell polarisation. Although type-1 and type-3 T_REG_ cells enhance the establishment of T_RM_ cells upon *Aspergillus* infection (Fig. 5), the absence of either type-1 or type-3 T_REG_ cells does not alter the polarisation toward the production of IFNγ or IL-17 (Fig. 6e, f).

Our data suggest that CD8^+^ T_RM_ cell formation occurs predominantly during type-1 immune responses, largely depending on type-1 T_REG_ cell recruitment and action. Type-3 infections do give rise to CD8^+^ T_RM_ cells as well, but these are more modest in numbers. Although there is preferential recruitment of infection-type-matching T_REG_ cells, there is redundancy between T_REG_ populations, especially when polarisation is less dominant. After establishment, CD8^+^ T_RM_ cells maintain their original profile, determined by the infection type, after pathogen clearance.

## Discussion

Non-lymphoid tissue immunity is important to guard against reinfection, local containment of invaders and to reduce the chance of systemic dissemination^34^. This is established after primary infection and is of great interest for vaccine strategies. T_REG_ cells are a critical component in the management of immune responses, maintaining self-tolerance and homoeostasis. Different populations of T_REG_ cells exist and co-localise with the corresponding CD4^+^ Th cell subsets, with similar transcriptional programmes and associated expression of chemokine receptors^27,35,36^. Our previous data showed the requirement of type-1 T_REG_ cell recruitment to the site of inflammation to release and make available TGFβ, thereby facilitating CD8^+^ T_RM_ cell development. The work focussed on an intestinal intracellular parasite infection, *Eimeria vermiformis*, invoking a type-1 dominated response with CD8^+^ T_RM_ cell development dependent on type-1, Tbet- and CXCR3-expressing, T_REG_ cells^14^. Mirroring of transcription programmes and chemokine receptor expression raised the question that particular T_REG_ subsets may control immune responses within the context of the pathogen encountered. This suggests that the development of CD8^+^ T_RM_ cells may require a match between type of infection and T_REG_ subset to be recruited^22^.

Localised infections result in strong CD8^+^ T_RM_ cell development, require inflammation-mediated lymphocyte trafficking and the cognate antigen to be present in the local microenvironment^14,34,37,38^. Our results are obtained using three distinct infection models in the lungs. In each of the models, CD4^+^ T cells are recruited to the site of inflammation, and each type of infection is dominated by the corresponding T_H_ cell subset, in numbers and proportions; Influenza by T_H_1 cells (IFNγ), *N. brasiliensis* by T_H_2 cells (IL-4, IL-13), and *A. fumigatus* by T_H_17 cells (IL-17). A potential limitation of our analysis is that we did not in vivo label the studied cells, a method used to identify circulating cells. However, this method has some disadvantages as it does not address migration properties and, in a highly vascular organ such as the lungs, the inflammatory context might result in tissue permeability and affect the results^39^.

We tested the role of type-3 T_REG_ cells in the establishment of CD8^+^ T_RM_ cells after a type-3 infection by genetically removing them using Foxp3^ΔRorγt^ animals. We successfully used this approach with Foxp3^ΔTbx21^ animals^14^. In Foxp3^ΔRorγt^ animals, the total number of T_REG_ cells, including type-1 T_REG_ cells that contribute to CD8^+^ T_RM_ cell development, T_H_17 cells, and CD8^+^ T cells expressing Rorγt were not altered. This confirms that the Foxp3-Cre-driven deletion is T_REG_-specific and attests to the careful genotyping of mice used. Conform our previous results in the small intestine^14^, predominantly type-1 T_REG_ cells are recruited upon a viral infection in the lung, which are required to enhance the establishment of CD8^+^ T_RM_ cells. CD8 T cells are also recruited to the site of the influenza infection and are nearly exclusively IFNγ-producers. Although we observe a robust T_H_2 cell response upon *N. brasiliensis* infection, and although more T_REG_ cells are recruited, we did not see a predominance of type-2 T_REG_ cells at the site of infection. In addition, CD8^+^ T cells are marginally recruited to, and activated at, the site of infection, although the presence of more endogenous CD8^+^ T_RM_ cells was observed. It is not clear if these cells are an expansion of the local CD8^+^ T_RM_ cell population responding to a local inflammation or if these are pathogen-specific. However, using CD45.1 adoptive transfers, it is clear that few naïve CD8^+^ T cells are recruited to the site of infection, while CD4^+^ T cells are. The cytokine expression profile of CD8^+^ T cells did not differ from non-infected controls, and the characteristic type-2 cytokines IL-4 and IL-13 were not detected. CD8^+^ T cells are not associated with type-2 or anti-helminth responses^40^. There are suggestions that helminths may themselves reduce the CD8^+^ T-cell response^41^. Therefore, our results may be applicable to helminth-induced type-2 responses but not represent allergic responses.

Type-3-dominated infections, represented by *A. fumigatus* and SFB, do result in preferential recruitment of type-3 T_REG_ cells as well as the recruitment of CD8^+^ T cells. However, and in contrast to CD4^+^ T cells, the CD8^+^ T cells recruited are less numerous compared to influenza. IL-17-producing CD8^+^ T cells are present after type-3 infection, but constitute a small population. CD8^+^ T_RM_ cell development does take place, but is not as efficient as observed during a type-1 response. In contrast to Foxp3^ΔTbx21^ animals in which we observe a marked decrease in CD8^+^ T_RM_ cells under steady-state conditions without known infection, Foxp3^ΔRorγt^ animals have numbers of CD8^+^ T_RM_ cells comparable to controls. This indicates that type-3 T_REG_ cell recruitment does not make a critical contribution to the overall development of CD8^+^ T_RM_ cells. Upon infection with *A. fumigatus* or SFBs, however, the absence of type-3 T_REG_ cells makes CD8^+^ T_RM_ cell development less efficient.

We confirm that the development of CD8^+^ T_RM_ cells during a type-1 infection, such as influenza, does not depend on type-3 T_REG_ cells but on type-1 T_REG_ cell recruitment exclusively. Less polarised responses or non-type-1 dominated infections are less dependent on specific T_REG_ subset recruitment to facilitate CD8^+^ T_RM_ cell development. This is in agreement with the T cells detected during the *A. fumigatus* or SFB infection showing a type-1 and type-3 identity, hence relying on type-1 and type-3 T_REG_ cell recruitment for CD8^+^ T_RM_ cell establishment. Infections are often a mixed response with type-1 responses playing an important role in most^42^. This may reflect the involvement of CD8^+^ T cells, which show an overwhelming type-1 dominated response. In accordance, the development of CD8^+^ T_RM_ cells during type-3 responses is not exclusively dependent on type-3 T_REG_ cell recruitment, but also on type-1 T_REG_ cells. However, the polarisation of CD8^+^ T_RM_ cells is determined by the infection and not by the recruitment of type-1 or type-3 T_REG_ cells. In the context of any invader, the establishment of a tissue-resident T-cell memory pool will be an advantage to locally contain the infection, independent of antigen specificity^43^.

The functional relevance of mismatched CD8^+^ T_RM_ cells during a reinfection is speculative but type-3 responses are flexible and can be converted to give rise to type-1 cells^44^. Although largely observed on CD4^+^ T cells, a mixed type-1/-3 response can be beneficial to reduce pathogen load and pathology in subsequent infections^45–49^. In addition, tissue repair can be enhanced by type-2 responses and type-3 CD8^+^ T_RM_ cells have been reported in the context of signals from injury and exposure to inflammatory mediators to release type-2 cytokines^50^. The physiological and clinical relevance of this mixed response, especially for CD8^+^ T_RM_ cells, is currently not clear but a subject for future investigation.

In summary, we extend previous work that T_REG_ cells, critical in preventing autoimmunity and immunopathology, have an important role in efficiently generating tissue-resident memory T cells from effector or memory precursors. We show that type-2 helminth infections are unlikely to result in robust de novo CD8^+^ T_RM_ cell formation. Type-3 infections result in CD8^+^ T_RM_ cell development, but less robust than type-1 infections. Type-1 T_REG_ cell recruitment is required for CD8^+^ T_RM_ cell development during type-1 infections, although type-3 T_REG_ cells may assist in less type-1 dominated responses, resulting in a predominantly IFNγ–producing CD8^+^ T_RM_ cell population. During type-3 infection, both type-1 and -3 T_REG_ cells play an important role, resulting in the establishment of a CD8^+^ T_RM_ cell population containing a mix of IFNγ- and IL-17-producing cells. Excluding helminths, independently of the type of pathogen encountered, CD8^+^ T_RM_ cells are generated, offering enhanced protection against future challenges.

## Methods

### Mice

C57BL/6 J CD45.1 mice were purchased from Charles River, France. Tbx21^fl/fl^ (Tbx21^tm2Srnr^) were kindly provided by Dr. Steven Reiner^51^, Foxp3eYFP-Cre (Foxp3^tm4(YFP/icre)Ayr^) was kindly provided by Dr. Alexander Rudensky^52^, Rosa26-tdRFP was kindly provided by Dr. Hans Jörg Fehling^53^. RORγt^fl/fl^ mice were obtained on Jackson Laboratories, Foxp3eYFP-Cre TGFβ1^-/-^ mice were kindly provided by Dr. Julien Marie^14^. Mice were bred at the Instituto de Medicina Molecular, Lisbon, Portugal. Male and female mice, aged and sex-matched, at 12–25 weeks of age were used. Animals were housed in IVC cages with temperature-controlled conditions under a 12-h light/dark cycle with free access to drinking water and food. All mice were kept in specific pathogen-free conditions. All mice in the Foxp3eYFP-Cre Rosa26-tdRFP lines were stringently genotyped by PCR and those in which a knockout allele was detected were discarded (~20%). In addition, mice were counter-screened for inappropriate expression of RFP in relation to eYFP (~10% discarded). All animal experimentation complied with regulations and guidelines of, and was approved by, the Direção-Geral de Alimentação e Veterinária Portugal and the local ethical review committee (Orbea).

### Cell isolation

ntestine was flushed with PBS to remove contents and opened longitudinally. After cutting into 1 cm pieces, it was incubated in PBS containing 20 mM Hepes, 100 U/ml penicillin, 100 µg/ml streptomycin, 1 mM Pyruvate, 10% FCS, 100 μg/ml polymyxin B and 10 mM EDTA for 30 min at 37 °C while shaking to release IELs. IEL single-cell suspensions were further purified using 37.5% isotonic Percoll. To isolate LPLs, intestinal tissue was then digested in IMDM medium containing 0.5 mg/ml of Collagenase D (Roche) and 0.2 mg/ml of DNaseI (Roche) for 25 min at 37 °C while shaking.

Lungs were perfused with 20 mL of PBS before being shredded in small pieces with scissors and digested in IMDM medium containing 5% FBS and 0.5 mg/ml Collagenase D, 37 °C during 30 min while shaking. The cell suspension containing the lymphocytes was passed through a 50 μm cell strainer, incubated for 2 min in ACK solution, and lymphocytes were obtained after a 6-min wash with PBS at 500 g centrifugation.

### Adoptive cell transfers

CD8α^+^ T cells and/or CD25^+^ cells (T_REG_ cells) were purified from a single-cell suspension of spleen and lymph nodes. Briefly, cells were labelled with anti-CD8a-APC or anti-CD25-APC antibodies and selected with anti-APC MACS microbeads, according to the manufacturer’s instructions. After counting, purity was determined by flow cytometry, and cell numbers were adjusted. To ensure a wide TCR diversity in the population transferred a minimum of 2 × 10^6^ CD8^+^ T cells were used. Some of the recipient mice received in addition 0.6–1 × 10^6^ T_REG_ cells. Infection was performed one day after cell transfer (day 0).

### Influenza X-31 preparation and infection

Reverse genetics A/X-31 (PR8-HK4 and 6) were used as model viruses. Reverse genetics derived chimeric PR8 containing the segment 4 from A/Hong Kong/1/1968, seg4-HK68 (PR8-HK4), or the segment 6 (PR8-HK6) were produced as previously described^54–56^. pDual plasmids were a kind gift from Dr. Ron Fouchier (Erasmus MC, Netherlands). PR8 NA-E229A^57^ was generated by reverse genetics after site-directed mutagenesis of pDual::segment 6. All viruses were amplified in embryonated chicken eggs and titrated using plaque assay as previously described^58,59^. A viral load of 1000 PFU was administered in 30 μL PBS intranasally to mice. Mouse weight was monitored daily during the experiment to certify welfare, with mice going lower than 25% initial weight being sacrificed.

### Eimeria vermiformis infection

Animals were infected with *Eimeria vermiformis* (Ev) as previously described in detail^33^. Briefly, oocysts were washed three times with deionized water, floated in sodium hypochloride and counted using a Fuchs-Rosenthal chamber. Mice received 5000 oocysts of *E. vermiformis* by oral gavage in 100 μl of water.

### *N. brasiliensis* infection

*N. brasiliensis* worms were propagated as previously described^30^. Infective L3 larvae were kindly provided by Brian Chan at Dr Judith E Allen’s laboratory. Briefly, the larvae were obtained by cutting the filter paper in which they were provided around the borders (~0.5 cm), emerging it around gauze in a Falcon tube with 40 mL of PBS at 37 °C for 10 min (Baermann’s method). After this, larvae were allowed to pool down by gravity for 10 min, with the bottom 10–15 mL containing them being transferred to a 15 mL tube. The larvae were then purified by performing three washes at 168 g with minimum break. Mice were then injected with 300 L3s subcutaneously.

### A. fumigatus preparation and infection

Fungal isolate *A. fumigatus* strain Af293 was purchased from the Fungal Genetics Stock Center (Kansas City, MO, United States). The fungal strain was cultivated on T-flasks containing sabouraud dextrose agar (SDA). Cultures were incubated in the dark, for 3–4 days, at 37 °C. Asexual spores (conidia) were harvested using glass beads and a saline solution B (0.85% NaCl, 0.1% Tween-20), washed twice with a saline solution A (0.85% NaCl), and collected after passing through three layers of miracloth filter. The harvested conidia were resuspended in a phosphate-buffered saline solution (0.01 M phosphate buffer, 0.0027 M potassium chloride, 0.137 M sodium chloride, pH 7.4), and kept at 4 °C until mice infection (same day). Prior to infection, conidia were counted with a hemacytometer and subsequently resuspended to a concentration of 3.3 × 10^7^ conidia/mL^−1^, 30 µL were used for mice infection (106 conidia). Mice were submitted to 4 intranasal challenges with 10^6^ conidia every 3 days over 2 weeks.

### SFB fecal transplant

SFB-containing feces were kindly provided by Dr. Gérard Eberl at Pasteur Institute, Paris. Fecal pellets were collected and kept at −20 °C to maintain integral microbiota. On the day of the fecal transplant, feces were defrosted, mashed, and suspended in filtered tap water at a concentration of 100 mg/mL. Mice then received a fecal transplant of 200 μL of this suspension by oral gavage.

### Flow cytometry

Single-cell suspensions from spleen, intestine, and lung were prepared and stained with antibodies (Supplementary Table 1), according to the agreed standards^60^. For intracellular cytokine staining, cells were pre-stimulated with PDBU (Phorbol 12,13-dibutyrate) (0.5 µg/mL) and ionomycin (0.5 μg/mL), in the presence of Brefeldin A (2 μg/mL) (all from Sigma) for 2 h at 37 °C. Samples were run on a Fortessa X20 cytometer (BD Biosciences) and analysed with FlowJo X software (TreeStar) (Supplementary Fig. 7).

### Statistical analysis

In the present work, statistical analyses were performed using GraphPad Prism Software (GraphPad Prism version 9 for Windows, GraphPad Software, San Diego, California USA), details described in each figure legend where applicable. *N* denotes the number of independent biological repeats and *n* the total number of samples (mice). Error bars represent S.E.M. Mann–Whitney analysis was applied to compare ranks between two groups with a *p* value of 0.05. *P < 0.05, **P < 0.01, ***P < 0.001.

### Reporting summary

Further information on research design is available in the Nature Portfolio Reporting Summary linked to this article.

### Supplementary information

Supplementary information

Reporting Summary

### Source data

Source data

## Data Availability

The authors declare that data supporting the findings of this study are available within the paper and its supplementary information files. Source data are provided with this paper. Additional materials or data that support the findings of this study are available from the corresponding authors. Source data are provided with this paper.
