# Supplementary material for: CD8+ tissue-resident memory T-cell development depends on infection-matching regulatory T-cell types
Source: Nat Commun. 2023 Sep 11;14:5579. doi: 10.1038/s41467-023-41364-w (PMC10495327; doi:10.1038/s41467-023-41364-w)
Supplement: Supplementary file 1 — Supplementary information [file 41467_2023_41364_MOESM1_ESM.pdf]

Supplementary data file

CD8$^+$ tissue-resident memory T cell development depends on infection matching regulatory T cell types

Leandro Barros[1], Daryna Piontkivska[2], Patrícia Figueiredo-Campos[1], Júlia Fanczal[1], Sofia Pereira Ribeiro[1], Marta Baptista[1], Silvia Ariotti[1], Nuno Santos[3,4], Maria João Amorim[3,5], Cristina Silva Pereira[2], Marc Veldhoen[1*#] and Cristina Ferreira[1*#]

[1]Instituto de Medicina Molecular | João Lobo Antunes, Faculdade de Medicina da Universidade de Lisboa, Av. Professor Egas Moniz, Lisbon, 1649-028, Portugal
[2]Instituto de Tecnologia Química e Biológica António Xavier, Av. da República, Oeiras, 2780-157 Portugal
[3]Instituto Gulbenkian de Ciência, Rua da Quinta Grande 6, Oeiras, 2780-156 Portugal
[4]Current address: The Francis Crick Institute, 1 Midland Road, London NW1 1AT, UK
[5]Universidade Católica Portuguesa, Católica Médical School, Católica Biomedical Research Centre, Palma de Cima, 1649-023, Portugal.

[*]These authors jointly supervised this work.

Supplementary data inventory

Supplementary data figures 1-7

Supplementary Figure 1. *Influenza* infection results in strong T cell response in the lungs

Supplementary Figure 2. *Nippostrongylus brasiliensis* infection results in a type-2 response, with limited CD8 T cell recruitment to the lungs

Supplementary Figure 3. *Aspergillus fumigatus* infection results in a type-3 response, with potented CD8 T cell contribution

Supplementary Figure 4. Successful RORγt T_{REG} cell ablation

Supplementary Figure 5. SFB faecal transplant results in a mixed Th-1/Th-17 immune response

Supplementary Figure 6. T_{RM} cell identity is maintained

Supplementary Figure 7. Flow cytometry analysis gating strategy

Supplementary table 1

Details of antibodies used in the study

Supplementary Figure 1

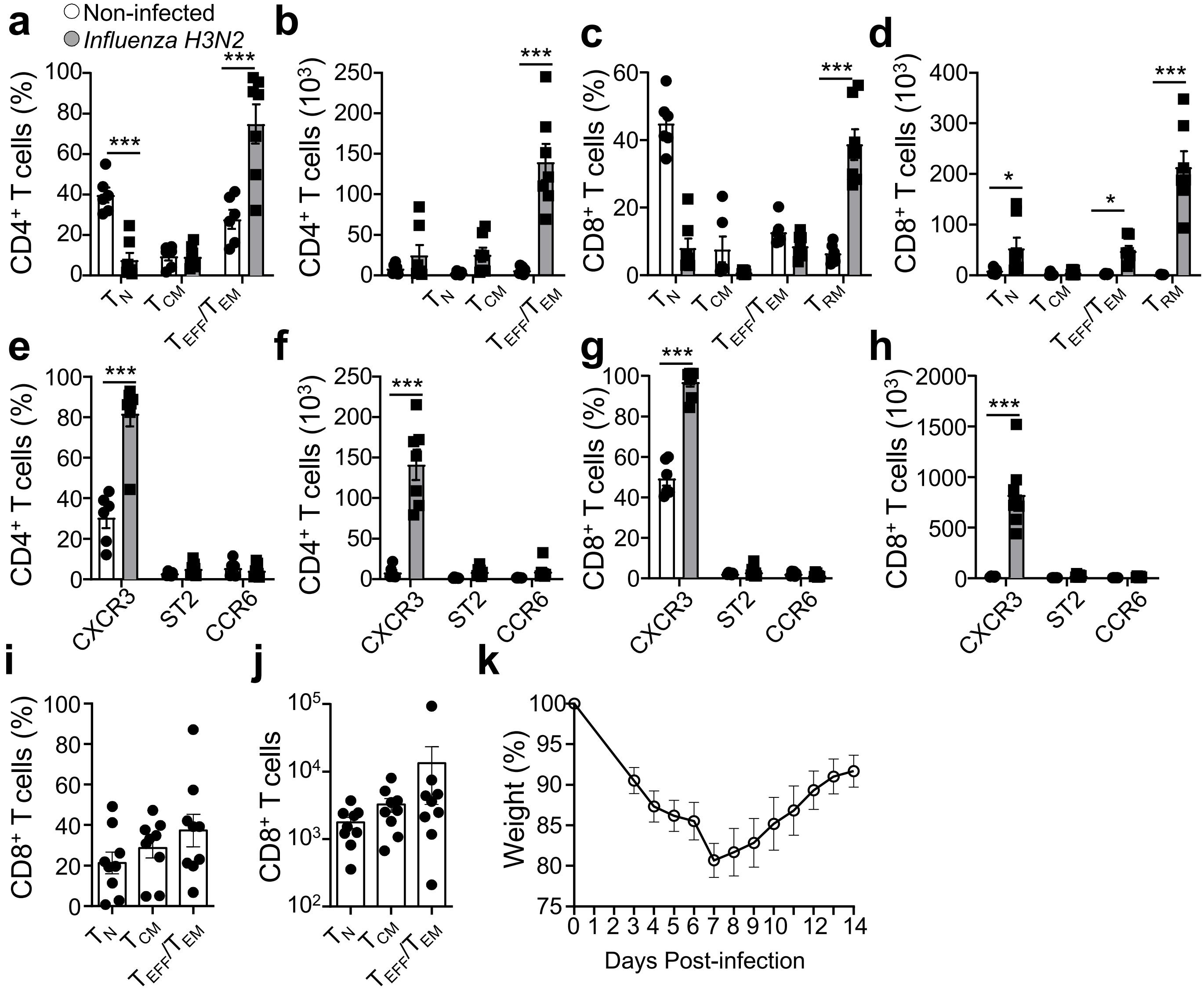

Supplementary Figure 1. *Influenza* infection results in strong T cell response in the lungs. C57BL/6 mice were intranasally infected with 1000 PFU of Influenza A X31 strain (H3N2). 10 days post-infection lungs were collected, and cells were isolated and analysed via flow cytometry for T cell subsets, with (a) percentage, $p_{(\text{Non-infected vs Influenza H3N2})}$: Tn, p=000119; Tem/Teff, p<0,000001) and (b) number, $p_{(\text{Non-infected vs Influenza H3N2})}$: Tem/Teff, p<0,000001, of $CD4^+$ T naïve (T_N $CD44^-CD62L^+$), central memory (T_{CM}, $CD44^+CD62L^+$) and effector memory/effector (T_{EM}/T_{EFF}, $CD44^+CD62L^-$) cells, and (c) percentage $p_{(\text{Non-infected vs Influenza H3N2})}$: T_{RM}, p<0,000001), and (d) number of $CD8^+$ T_N, T_{CM}, and T_{EM}/T_{EFF} cells with $CD8^+$ T_{RM} cells ($CD69^+KLRG1^-CD8^+$), $p_{(\text{Non-infected vs Influenza H3N2})}$: Tn, p=0,047002; Tem/Teff, p=0,040349;T_{RM}, p<0,000001) (non-infected n=6, infected n=7, N=3). (e,f,g,h). $Foxp3^{WT}$ mice were analysed for indicated receptors on (e,f) $CD4^+$ or (g,h) $CD8^+$ T cells in percentage (e,g) and numbers (f,h), $p_{(\text{Non-infected vs Influenza H3N2})}$: percentage and cell number CXCR3+ CD4 and CD8 T cells, p<0,000001, (non-infected n=6, infected n=8, N=3). (i,j) $Foxp3^{WT}$ mice received $CD8^{CD45.1}$ T cells intravenously, one day prior to infection: day 14 days post-infection the transferred T cells were assessed for their status in (i) percentage and (j) numbers of T_N, T_{CM} and T_{EM}/T_{EFF} cells in the spleen (n=9, N=4). (k) Representative weight curve after infection at day 0 (n=6, N=2). 2-sided Mann-Whitney analysis was applied to compare the differences between infected and non-infected groups. Data is presented as bars of mean ± SEM with single data points.

Supplementary Figure 2

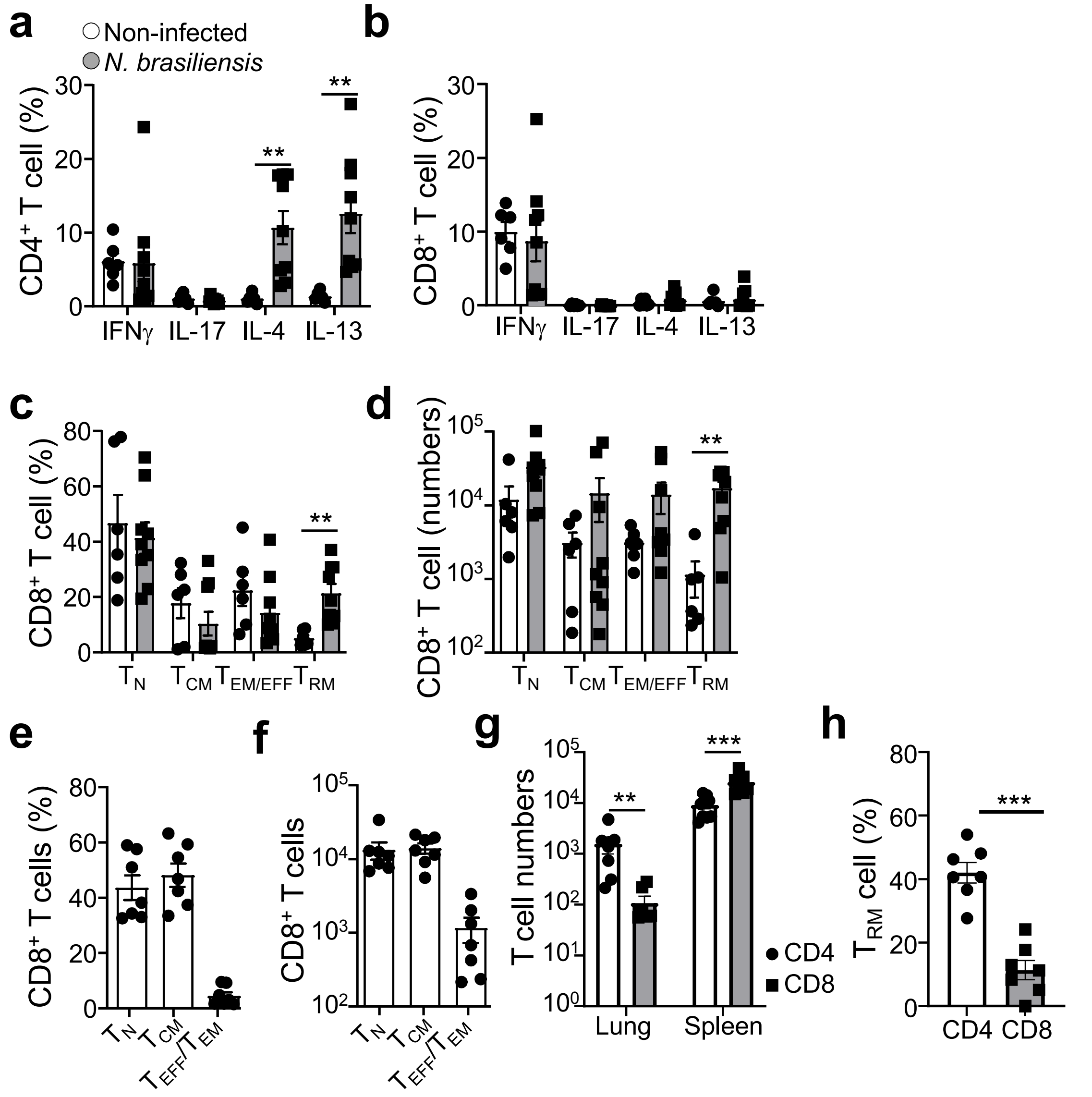

Supplementary Figure 2. *Nippostrongylus brasiliensis* infection results in a type-2 response, with limited CD8 T cell recruitment to the lungs. C57BL/6 mice were subcutaneously infected with 300 stage L3 larvae of *Nippostrongylus brasiliensis*. 7 days post-infection lungs were collected, and cells were isolated and analysed via flow cytometry for cytokine production and T cell populations, with percentage of $CD44^+$ (a) $CD4^+$, $p_{(\text{Non-infected vs } \textit{Nippostrongylus brasiliensis})}$: IL-4, p=0,004384; IL-13, p=0,004416 and (b) $CD8^+$ T cells producing indicated cytokines. (c,d) Analysis of $CD8^+$ T cells with (c) percentage, $p_{(\text{Non-infected vs } \textit{Nippostrongylus brasiliensis})}$: T_{RM}, p=0,002496), and (d) number, $p_{(\text{Non-infected vs } \textit{Nippostrongylus brasiliensis})}$: T_{RM}, p=0,003648) of $CD8^+$ T naïve (T_N $CD44^-CD62L^+$), central memory (T_{CM}, $CD44^+CD62L^+$), effector memory/effector (T_{EM}/T_{EFF}, $CD44^+CD62L^-$) cells, and tissue resident (T_{RM}, $CD69^+KLRG1^-$) cells (non-infected n=6, infected n=9, N=3). (e,f) $Foxp3^{WT}$ mice received $CD8^{CD45.1}$ T cells intravenously, one day prior to infection. At day 14 post-infection, spleens were analysed for presence of transferred cells in (e) percentage and (f) numbers of T_N, T_{CM} and T_{EM}/T_{EFF} cell subsets (n=7, N=3). (g,h) $Foxp3^{WT}$ mice received $CD8^{CD45.1}$ (n=7, N=3) or $CD4^{CD45.1}$ T cells (n=7 for lungs, n=9 for spleens, N=2) intravenously, one day prior to infection. At day 14 post-infection, spleens and lungs were analysed for presence of transferred cells (g), $p_{(\text{CD4 vs CD8})}$: Lung, p=0,002331; Spleen, p=0,000350) and the proportion of T_{RM} cells in the lungs (h), $p_{(\text{CD4 vs CD8})}$=0,0006. 2-sided Mann-Whitney analysis was applied to compare non-infected versus infected groups. Data is presented as bars of mean ± SEM with single data points.

Supplementary Figure 3

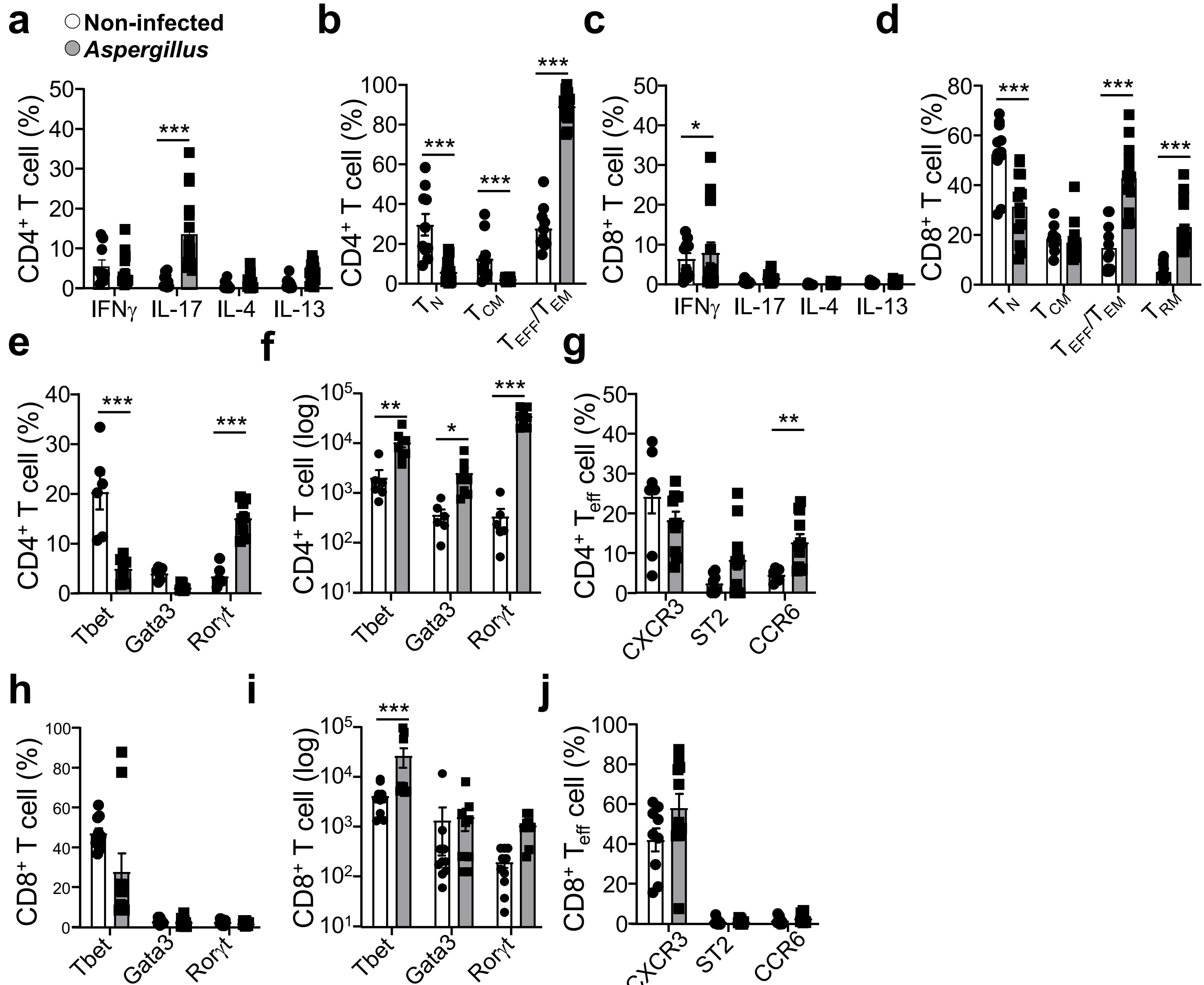

Supplementary Figure 3. *Aspergillus fumigatus* infection results in a type-3 response, with potented CD8 T cell contribution. C57BL/6 mice intranasally challenged four times every 3 days with 10^6 spores of *Aspergillus fumigatus*. 10 days post-infection lungs were collected, and cells were isolated and analysed via flow cytometry for cytokine production, and memory subsets. (a,b,c,d) Percentages of $CD4^+$ T cells with respect to (a) indicated cytokine production from $CD44^+$ cells, $p_{(\text{Non-infected vs } \textit{Aspergillus})}$: IL-17, p=0,000137, and (b) naïve (T_N, $CD44^-CD62L^+$), central memory (T_{CM}, $CD44^+CD62L^+$), effector memory/effector (T_{EM}/T_{EFF}, $CD44^+CD62L^-$) cells, $p_{(\text{Non-infected vs } \textit{Aspergillus})}$: Tn, p<000001; Tcm, p=0,017999; Tem/Teff, p<0,000001, or $CD8^*$ T cells, and (c) cytokine production from $CD44^+$ T cells, $p_{(\text{Non-infected vs } \textit{Aspergillus})}$: IFN-γ, p=0,044046 and (d) subset distribution of T_N, T_{CM}, T_{EM}/T_{EFF} and tissue resident memory (T_{RM}, $CD69^+KLRG1^-$) T cells, $p_{(\text{Non-infected vs } \textit{Aspergillus})}$: Tn, p=001575; Tem/Teff, p=0,000019; T_{RM}, p=0,000023, (cytokine production; non-infected, n=10; infected n=12, N=4; subset analysis: non-infected, n=10; infected n=13, N=4). (e,f,g,h,i,j) $Foxp3^{WT}$ mice were analysed for indicated major transcription factors in $CD4^+$ T cells, in (e) percentage, $p_{(\text{Non-infected vs } \textit{Aspergillus})}$: Tbet, p=0,000342; RORγt, p=0,000011, and (f) numbers, $p_{(\text{Non-infected vs } \textit{Aspergillus})}$: Tbet, p=0,008197; GATA-3, p=0,027401,RORγt, p=0,000029 (non-infected n=4, infected, n=5, N=3), $CD8^+$ T cells, in (h) percentage and (i) numbers, $p_{(\text{Non-infected vs } \textit{Aspergillus})}$: Tbet, p=0,000988 (non-infected n=8, infected, n=9, N=3), and chemokine receptors on (g) $CD4^+$, $p_{(\text{Non-infected vs } \textit{Aspergillus})}$: CCR6, p=0,003572 (non-infected n=6, infected n=11, N=3) and (j) $CD8^+$ T cells (non-infected n=7, infected n=8, N=3). 2-sided Mann-Whitney analysis was applied to compare the differences between non-infected and infected groups. Data is presented as bars of mean ± SEM with single data points.

Supplementary Figure 4

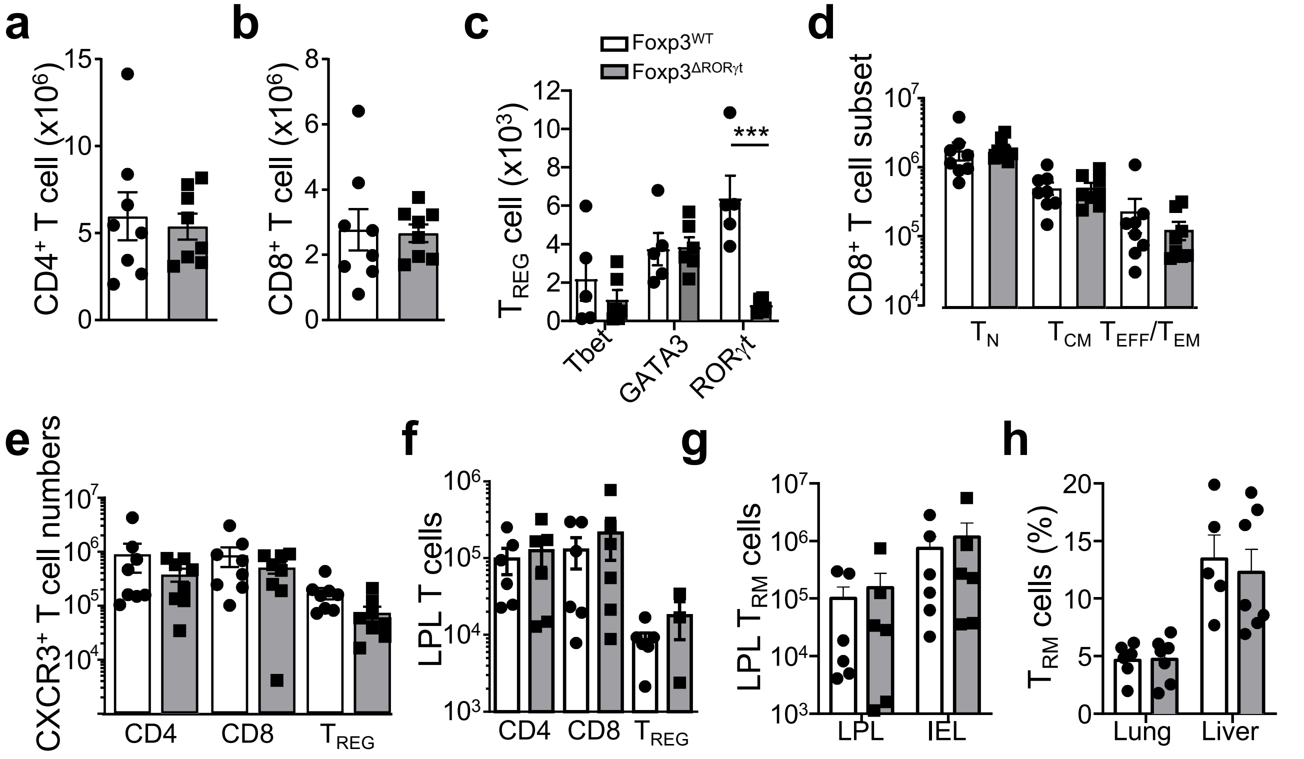

Supplementary Figure 4. Successful RORγt T_{REG} cell ablation. Foxp3[WT] and Foxp3[ΔRORγt] were compared at steady-state for their T_{REG}, $CD4^+$ and $CD8^+$ T cell compartments in lung, liver, spleen, small intestinal lamina propria lymphocytes (LPL), and small intestinal intraepithelial lymphocytes (IEL). (a,b) Total numbers of (a) $CD4^+$ and (b) $CD8^+$ T cells in spleen (n=8, N=3). (c) Assessment of LPL T_{REG} cell numbers for indicated transcription factors, $p_{(Foxp3WT\ vs\ Foxp3\Delta ROR\gamma t)}$<0,000001(Foxp3[WT] n=5, Foxp3[ΔRORγt] n=6, N=3). (d) Splenic $CD8^+$ T cell subset numbers for naïve (T_N, $CD44^-CD62L^+$), central memory (T_{CM}, $CD44^+CD62L^+$), effector memory/effector (T_{EM}/T_{EFF}, $CD44^+CD62L^-$) cells (n=8). (e) Cell numbers of chemokine receptor CXCR3-expressing CD4, CD8 and T_{REG} cells (n=8, N=3). (f) LPL $CD4^+$, $CD8^+$ and T_{REG} cell numbers (Foxp3[WT] n=6, Foxp3[ΔRORγt] n=7, N=3). (g) LPL and IEL T_{RM} ($CD69^+KLRG1^-CD103^+CD8^+$) cell number (n=6, N=3). (h) Assessment of lung and liver T_{RM} cell ($CD69^+KLRG1^-$ $CD8^+$) percentage within total $CD8^+$ T cells in both mouse lines (Foxp3[WT] n=5, Foxp3[ΔRORγt] n=6, N=3). 2-sided Mann-Whitney analysis was applied to compare the differences between mouse strains. Data is presented as bars of mean ± SEM with single data points.

Supplementary Figure 5

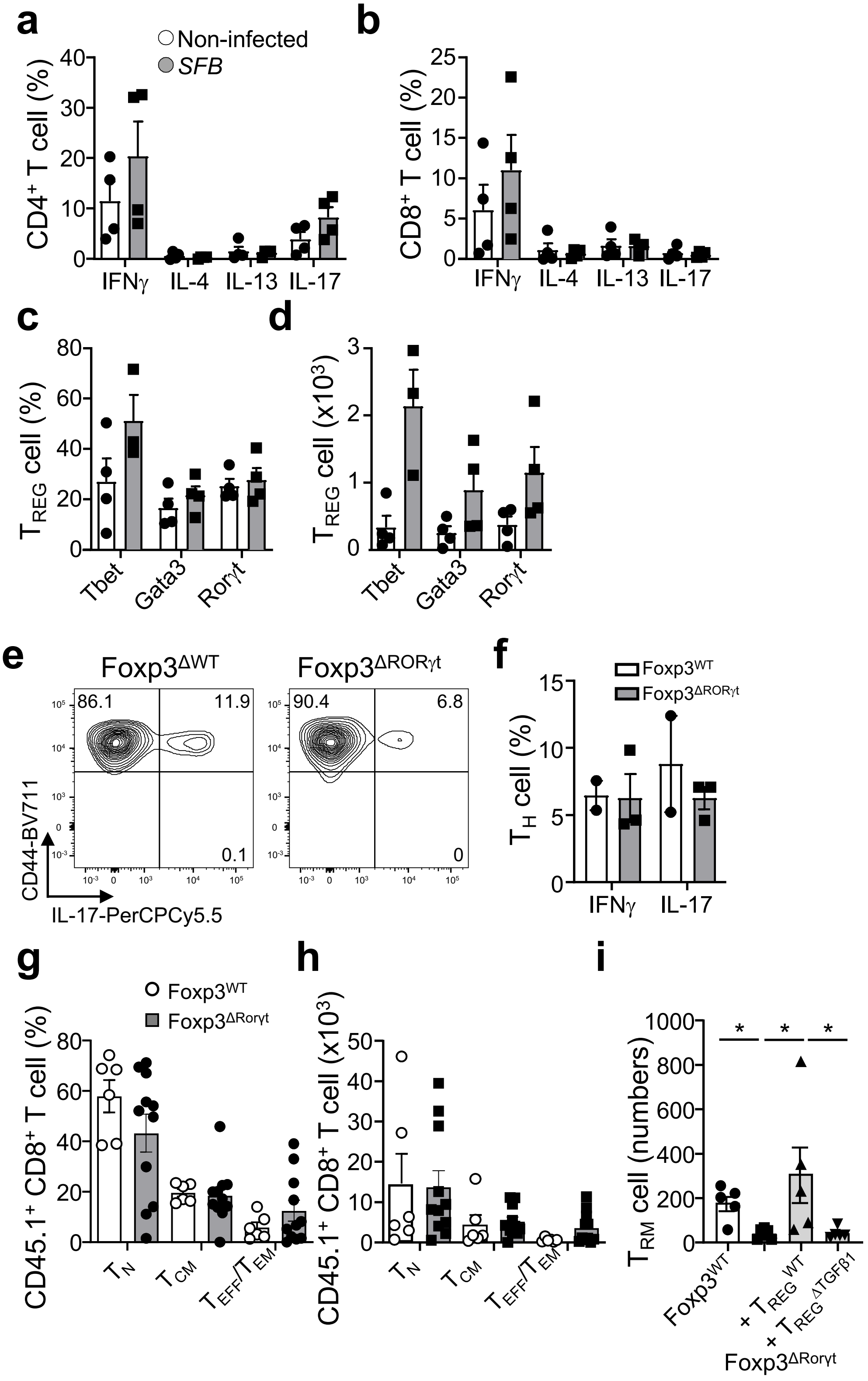

Supplementary Figure 5. SFB faecal transplant results in a mixed Th-1/Th-17 immune response. C57BL/6 mice received a transplant of 100mg of SFB-containing faeces. 10 days post-infection small intestine LPL were isolated and compared with the ones from non-infected mice via flow cytometry for cytokine production T_{REG} phenotypes. (a) Percentage of activated $CD44^{+}$ $CD4^{+}$ T cells producing indicated cytokines and (b) respective cell numbers (n=4, N=2). (c) Percentage of $Foxp3^{+}CD4^{+}$ T cells producing indicated transcription factors and (d) respective cell numbers (n=4, N=2). (e-f) $Foxp3^{WT}$ and $Foxp3^{\Delta ROR\gamma t}$ mice received the faecal transplant. Small intestine LPL were assessed for IFNγ and IL-17 production within their Th cell population. (e) Example flow plot for IFNγ and IL-17 within activated $CD4^{+}$ T cells and (f) respective percentage ($Foxp3^{WT}$ n=2, $Foxp3^{DRORgt}$ n=3, N=2). (g-h) Splenocytes were collected and analysed for the presence and memory phenotype of transferred cells. CD45.1 $CD8^{+}$ T cell (g) proportions in the spleen were assessed for naïve (T_N $CD44^{-}CD62L^{+}$), central memory (T_{CM}, $CD44^{+}CD62L^{+}$) and effector memory/effector (T_{EM}/T_{EFF}, $CD44^{+}CD62L^{-}$) and for their (h) respective numbers ($Foxp3^{WT}$ n=6, $Foxp3^{\Delta RORgt}$ n=11, N=3). i) Mice were transferred with indicated condition receiving control T_{REG} cells prior to the first of four intranasal challenges with 10^{6} spores of *Aspergillus fumigatus*. Lungs were collected and transferred CD45.1 CD8 T cells were analysed. Numbers of CD45.1 CD8 T_{RM} cells in all conditions, $p_{(Foxp3WT\ vs\ Foxp3\Delta ROR\gamma t)}$=0,0173, $p_{(Foxp3\Delta ROR\gamma t\ vs\ Foxp3\Delta ROR\gamma t+WT\ Tregs)}$=0,0108, $p_{(Foxp3\Delta ROR\gamma t\ vs\ Foxp3\Delta ROR\gamma t+Treg\Delta TGF\beta 1)}$=0,0303 ($Foxp3^{WT}$; $Foxp3^{\Delta ROR\gamma t}$ n=6, N=4; $Foxp3^{\Delta ROR\gamma t}$ + $T_{REG}{}^{WT}$ n=5, N=2; $Foxp3^{\Delta ROR\gamma t}$ + $T_{REG}{}^{\Delta TGF\beta 1}$ n=6, N=2). 2-sided Mann-Whitney analysis was applied to compare the differences between mouse strains. Data is presented as bars of mean ± SEM with single data points.

Supplementary Figure 6

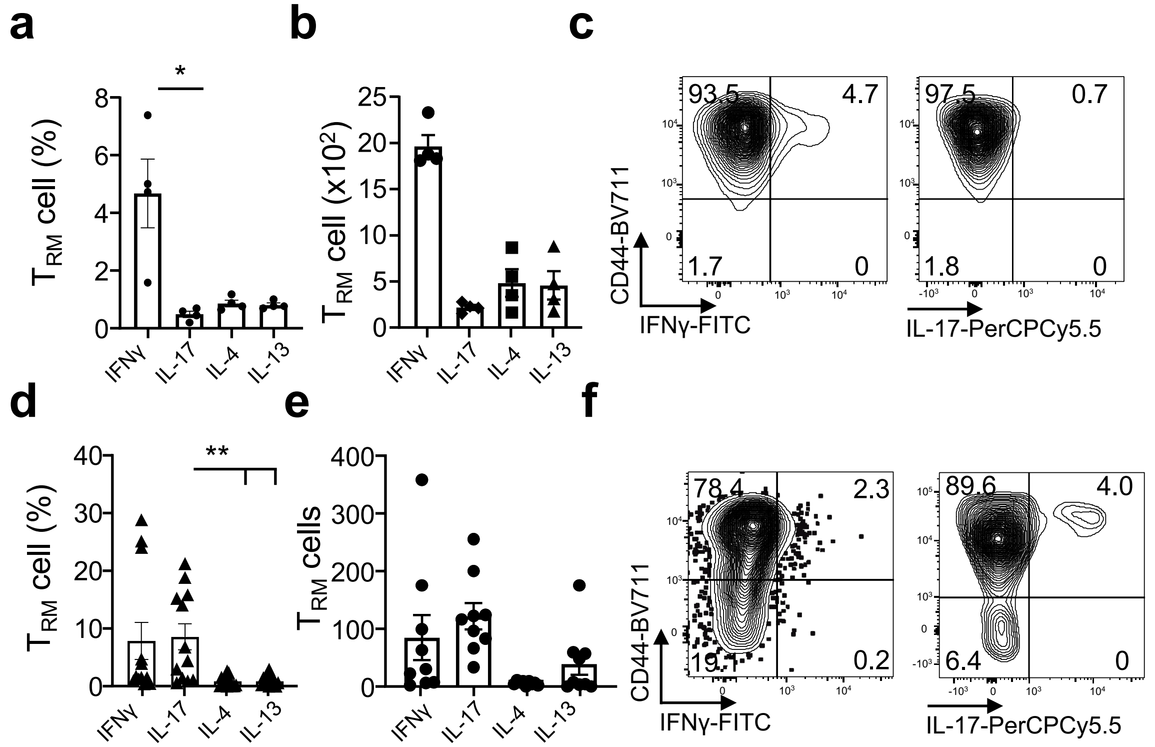

Supplementary Figure 6. T_{RM} cell identity is maintained. (a-c) Mice were infected intranasally with 1000 PFU of Influenza X31 strain (H3N2). Post-infection lungs were collected, and cells analysed. (a) Percentage of T_{RM} cells ($CD69^+KLRG1^-CD8^+$) producing indicated cytokines, $p_{(IFN\text{-}\gamma \text{ vs IL-17})}$=0,0286 and (b) respective numbers (n=4, N=2) and (c) representative flow cytometry plots for IFNγ and IL-17. (d-f) Mice were challenged four times with 10^6 spores of *Aspergillus fumigatus*, post-infection lungs were collected, and cells were analysed with flow cytometry for cytokine production. (d) Percentage of T_{RM} cells ($CD69^+KLRG1^-CD8^+$) producing indicated cytokines, $p_{(IL\text{-}17 \text{ vs IL-4})}$=0,0014 and $p_{(IL\text{-}17 \text{ vs IL-}13)}$=0,0011 (n=12, N=4), and (e) respective numbers (n=9, N=4) and (f) representative flow cytometry plots for IFNγ and IL-17. 2-sided Mann-Whitney analysis was applied to compare groups. Data is presented as bars of mean ± SEM with single data points.

Supplementary Figure 7

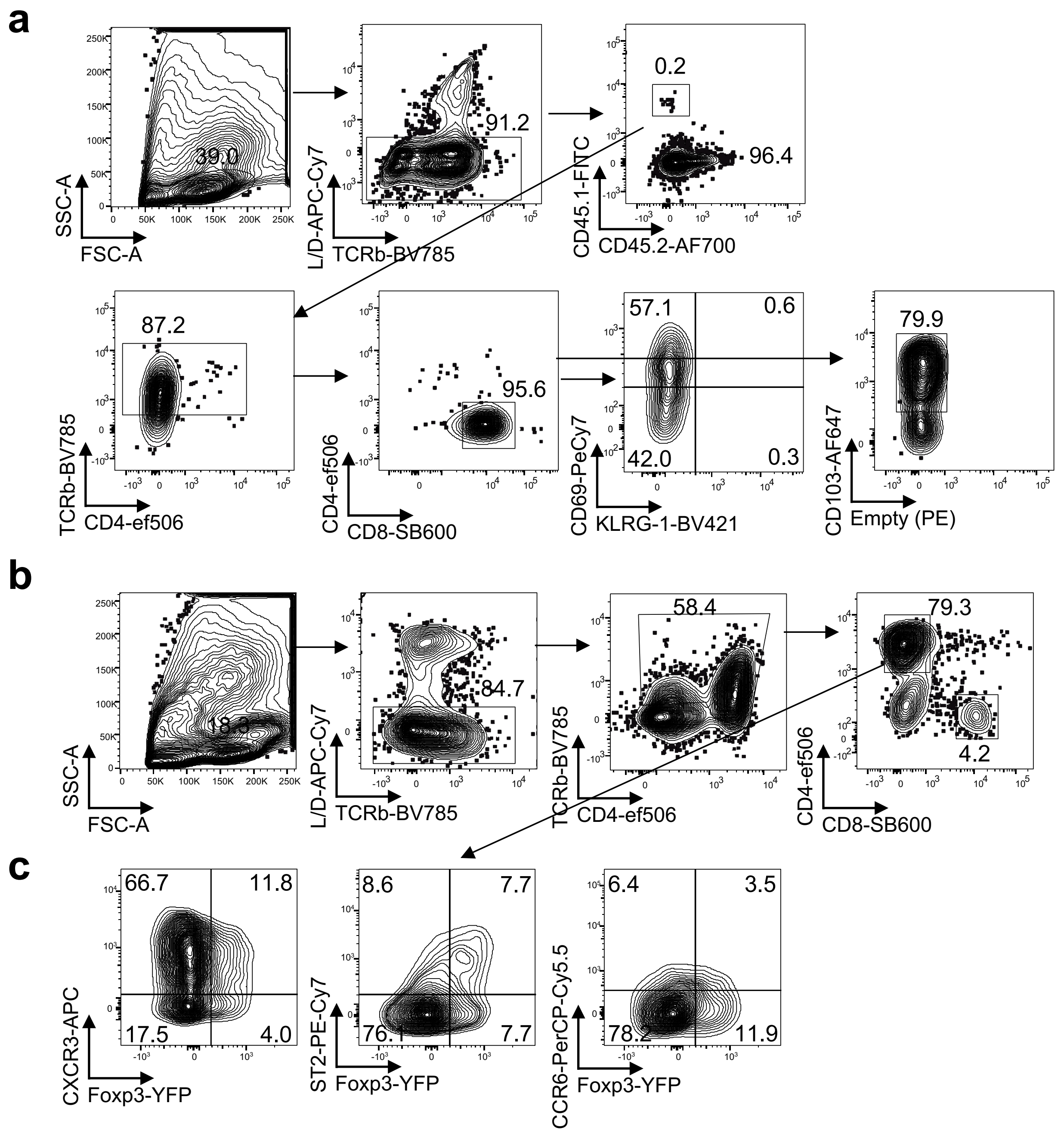

Supplementary Figure 7. Flow cytometry analysis gating strategy. a) Representative flow cytometry plots from lungs cells of a C57BL/6 mouse showing Lymphocyte gating; dead cell exclusion, followed by CD45.1 cell selection (if used): followed by T cell selection; followed by CD8 T cell selection and an example of CD69 - KLRG-1 or CD103 plotting. b) Representative flow cytometry plots from lung cells of a C57BL/6 mouse, showing Lymphocyte gating; dead cell exclusion: followed by T cell selection; followed by CD4 (or CD8) T cell selection. c) Example of T_{REG} cell and chemokine receptor staining using Foxp3-YFP, pre-gated on CD4.

For cytokine staining, pre-gated on CD4 or CD8, see main figures 1, 2 and 3.

Supplementary Table 1. Details of antibodies used in the study.

Epitope	Fluorochrome	Catalogue number	Clone	Dilution	Supplier
CD103	BV711	121435	2E7	1:300	Biolegend
	AF647	121410			
CD183 (CXCR3)	APC	126512	CXCR3-173	1:500	
CD196 (CCR6)	PerCPCy5.5	129810	29-2L17	1:200	
	PE	129804			
CD25	PerCP/Cy5.5	102028	PC61	1:500	
	APC	102012			
CD4	PE-Cy7	100528	RM4-5	1:1000	
	AF700	100430	GK1.5	1:800	
CD44	AF700	103026	IM7	1:500	
	BV711	103057			
CD45.1	PacificB	110722	A20	1:500	
	FITC	110706			
	BV605	110738			
CD45.2	AF647	109814	104	1:500	
	AF700	109822			
CD62L	FITC	104406	MEL-14	1:500	
	PE	104408			
CD69	PE-Cy7	104512	H1.2F3	1:300	
	BV650	104541			
CD8α	APC	100712	53-6.7	1:500	
	BV711	100748			
FoxP3	AF488	126406	MF-14	1:200	
KLRG1 (MAFA)	BV421	138414	2F1/KLRG1	1:1000	
ST2 (IL-33R)	PECy7	146610	DIH9	1:200	
T-bet	PECy7	644824	4B10	1:200	
IFNγ	APC	505810	XMG1.2	1:300	
	FITC	505806			
IL-4	PE	504104	11B11	1:200	
IL-17A	PerCPCy5.5	506920	TC11-18H10.1	1:300	
TCRβ	Pacific Blue	109226	H57-597	1:500	
	PerCPCy5.5	109228			
	BV785	109249			
CD4	ef506	69-0042-82	RM4-5	1:800	eBioscience
CD8α	SB600	63-0691-82	53-6.7	1:500	
Eomes	AF488	53-4875-80	DAN11MAG	1:200	
GATA-3	PE	14-9966-82	TWAJ	1:50	
RORγt	PE	12-6981-82	B2D	1:100	
	APC	17-6981-82			
IL-13	PE Cy7	25-7133-82	eBio13A	1:200	
