# Supplementary material for: CD8+ tissue-resident memory T-cell development depends on infection-matching regulatory T-cell types
Source: Nat Commun. 2023 Sep 11;14:5579. doi: 10.1038/s41467-023-41364-w (PMC10495327; doi:10.1038/s41467-023-41364-w)
Supplement: Supplementary file 2 — Reporting Summary [file 41467_2023_41364_MOESM2_ESM.pdf]

nature portfolio

Corresponding author(s): Cristina Ferreira and Marc Veldhoen

Last updated by author(s): 2023/08(16

Reporting Summary

Nature Portfolio wishes to improve the reproducibility of the work that we publish. This form provides structure for consistency and transparency in reporting. For further information on Nature Portfolio policies, see our Editorial Policies and the Editorial Policy Checklist.

Statistics

For all statistical analyses, confirm that the following items are present in the figure legend, table legend, main text, or Methods section.

n/a	Confirmed	
☐	☒	The exact sample size (*n*) for each experimental group/condition, given as a discrete number and unit of measurement
☐	☒	A statement on whether measurements were taken from distinct samples or whether the same sample was measured repeatedly
☐	☒	The statistical test(s) used AND whether they are one- or two-sided<br>*Only common tests should be described solely by name; describe more complex techniques in the Methods section.*
☒	☐	A description of all covariates tested
☒	☐	A description of any assumptions or corrections, such as tests of normality and adjustment for multiple comparisons
☐	☒	A full description of the statistical parameters including central tendency (e.g. means) or other basic estimates (e.g. regression coefficient) AND variation (e.g. standard deviation) or associated estimates of uncertainty (e.g. confidence intervals)
☐	☒	For null hypothesis testing, the test statistic (e.g. *F*, *t*, *r*) with confidence intervals, effect sizes, degrees of freedom and *P* value noted<br>*Give P values as exact values whenever suitable.*
☒	☐	For Bayesian analysis, information on the choice of priors and Markov chain Monte Carlo settings
☒	☐	For hierarchical and complex designs, identification of the appropriate level for tests and full reporting of outcomes
☒	☐	Estimates of effect sizes (e.g. Cohen's *d*, Pearson's *r*), indicating how they were calculated

Our web collection on statistics for biologists contains articles on many of the points above.

Software and code

Policy information about availability of computer code

Data collection	Flow cytometry collection: BD FACSDiva v9.0 Software
Data analysis	Flowjo version X was used for all flow cytometry data analysis.

For manuscripts utilizing custom algorithms or software that are central to the research but not yet described in published literature, software must be made available to editors and reviewers. We strongly encourage code deposition in a community repository (e.g. GitHub). See the Nature Portfolio guidelines for submitting code & software for further information.

Data

Policy information about availability of data

All manuscripts must include a data availability statement. This statement should provide the following information, where applicable:

- Accession codes, unique identifiers, or web links for publicly available datasets
- A description of any restrictions on data availability
- For clinical datasets or third party data, please ensure that the statement adheres to our policy

The authors declare that data supporting the findings of this study are available within the paper and its supplementary information files. The source data are provided with this paper. Additional data that support the findings of this study are available from the corresponding authors.

Human research participants

Policy information about studies involving human research participants and Sex and Gender in Research.

Reporting on sex and gender	N/A
Population characteristics	N/A
Recruitment	N/A
Ethics oversight	N/A

Note that full information on the approval of the study protocol must also be provided in the manuscript.

Field-specific reporting

Please select the one below that is the best fit for your research. If you are not sure, read the appropriate sections before making your selection.

- [x] Life sciences
- [ ] Behavioural & social sciences
- [ ] Ecological, evolutionary & environmental sciences

For a reference copy of the document with all sections, see nature.com/documents/nr-reporting-summary-flat.pdf

Life sciences study design

All studies must disclose on these points even when the disclosure is negative.

Sample size	Sample size was determined based on the complexity and cost of the experiments as well as availability of mice with the desired genotype.
Data exclusions	T cell transfer studies in which less than 70 cells were recovered were excluded from further analysis.
Replication	All experiments include multiple samples and each experiment was repeated at least twice, mostly three times. Most data of repeats were included in the manuscript. The number of experiments performed for each panel is indicated in Figure legend.
Randomization	No randomization of groups was performed. All mice were distributed according to the genotype required. The animals were all inoculated with same concentration and under the same conditions with each pathogen and all analysed, therefore randomization was not necessary in this study.
Blinding	No blinding of groups was perfomed. Authors infected all groups under the same conditions with the same stock, data acquired did not depend on non-objective scoring methods, but acquisition of data not effected by blinding. Hence, blinding was not introduced.

Reporting for specific materials, systems and methods

We require information from authors about some types of materials, experimental systems and methods used in many studies. Here, indicate whether each material, system or method listed is relevant to your study. If you are not sure if a list item applies to your research, read the appropriate section before selecting a response.

Materials & experimental systems

n/a	Involved in the study	
[ ]	[x]	Antibodies
[x]	[ ]	Eukaryotic cell lines
[x]	[ ]	Palaeontology and archaeology
[ ]	[x]	Animals and other organisms
[x]	[ ]	Clinical data
[x]	[ ]	Dual use research of concern

Methods

n/a	Involved in the study	
[x]	[ ]	ChIP-seq
[ ]	[x]	Flow cytometry
[x]	[ ]	MRI-based neuroimaging

Antibodies

Antibodies used

Antigen Fluorochrome Clone Supplier #cat Validation / TDS

CD103 BV711 2E7 Biolegend 121435 https://www.biolegend.com/en-us/products/brilliant-violet-711-anti-mouse-cd103-antibody-14411?pdf=true&displayInline=true&leftRightMargin=15&topBottomMargin=15&filename=Brilliant%20Violet%20711™%20anti-mouse%

20CD103%20Antibody.pdf

CD103 AF647 2E7 Biolegend 121410 https://www.biolegend.com/en-us/global-elements/pdf-popup/alexa-fluor-647-anti-mouse-cd103-antibody-3576?filename=Alexa Fluorreg 647 anti-mouse CD103 Antibody.pdf&pdfgen=true

CD183 (CXCR3) APC CXCR3-173 Biolegend 126512 https://www.biolegend.com/en-us/global-elements/pdf-popup/apc-anti-mouse-cd183-cxcr3-antibody-4683?filename=APC anti-mouse CD183 CXCR3 Antibody.pdf&pdfgen=true

CD196 (CCR6) PerCP/Cy5.5 29-2L17 Biolegend 129810 https://www.biolegend.com/en-us/global-elements/pdf-popup/percp-cyanine5-5-anti-mouse-cd196-ccr6-antibody-5223?filename=PerCPCyanine55 anti-mouse CD196 CCR6 Antibody.pdf&pdfgen=true

CD196 (CCR6) PE 29-2L17 Biolegend 129804 https://www.biolegend.com/en-us/products/pe-anti-mouse-cd196-ccr6-antibody-5220?pdf=true&displayInline=true&leftRightMargin=15&topBottomMargin=15&filename=PE%20anti-mouse%20CD196%20(CCR6)%20Antibody.pdf

CD25 PerCP/Cy5.5 PC61 Biolegend 102028 https://www.biolegend.com/en-us/products/percp-anti-mouse-cd25-antibody-4263?pdf=true&displayInline=true&leftRightMargin=15&topBottomMargin=15&filename=PerCP%20anti-mouse%20CD25%20Antibody.pdf

CD25 APC PC61 Biolegend 102012 https://www.biolegend.com/en-us/products/apc-anti-mouse-cd25-antibody-420?pdf=true&displayInline=true&leftRightMargin=15&topBottomMargin=15&filename=APC%20anti-mouse%20CD25%20Antibody.pdf

CD4 PE-Cy7 RM4-5 Biolegend 100528 https://www.biolegend.com/en-us/global-elements/pdf-popup/pe-cy7-anti-mouse-cd4-antibody-1932?filename=PECy7 anti-mouse CD4 Antibody.pdf&pdfgen=true

CD4 AF700 GK1.5 Biolegend 100430 https://www.biolegend.com/en-us/global-elements/pdf-popup/alexa-fluor-700-anti-mouse-cd4-antibody-3385?filename=Alexa Fluorreg 700 anti-mouse CD4 Antibody.pdf&pdfgen=true

CD4 ef506 RM4-5 eBioscience 69-0042-82 https://www.thermofisher.com/order/genome-database/dataSheetPdf?producttype=antibody&productsubtype=antibody_primary&productId=69-0042-82&version=251

CD44 A700 IM7 Biolegend 103026 https://www.biolegend.com/en-us/global-elements/pdf-popup/alexa-fluor-700-anti-mouse-human-cd44-antibody-3406?filename=Alexa Fluorreg 700 anti-mousehuman CD44 Antibody.pdf&pdfgen=true

CD44 BV711 IM7 Biolegend 103057 https://www.biolegend.com/en-us/products/brilliant-violet-711-anti-mouse-human-cd44-antibody-10316?pdf=true&displayInline=true&leftRightMargin=15&topBottomMargin=15&filename=Brilliant%20Violet%20711™%20anti-mouse/human%20CD44%20Antibody.pdf

CD45.1 PacificB A20 Biolegend 110722 https://www.biolegend.com/en-us/global-elements/pdf-popup/pacific-blue-anti-mouse-cd45-1-antibody-3105?filename=Pacific Bluetrade anti-mouse CD451 Antibody.pdf&pdfgen=true

CD45.1 FITC A20 Biolegend 110706 https://www.biolegend.com/en-us/global-elements/pdf-popup/fitc-anti-mouse-cd45-1-antibody-198?filename=FITC anti-mouse CD451 Antibody.pdf&pdfgen=true

CD45.1 BV605 A20 Biolegend 110738 https://d1spbj2x7qk4bg.cloudfront.net/en-ie/products/brilliant-violet-605-anti-mouse-cd45-1-antibody-7850?pdf=true&displayInline=true&leftRightMargin=15&topBottomMargin=15&filename=Brilliant%20Violet%20605™%20anti-mouse%20CD45.1%20Antibody.pdf&v=20230524063105

CD45.2 AF647 104 Biolegend 109814 https://www.biolegend.com/en-us/global-elements/pdf-popup/apc-anti-mouse-cd45-2-antibody-2759?filename=APC anti-mouse CD452 Antibody.pdf&pdfgen=true

CD45.2 AF700 104 Biolegend 109822 https://www.biolegend.com/en-us/global-elements/pdf-popup/alexa-fluor-700-anti-mouse-cd45-2-antibody-3393?filename=Alexa Fluorreg 700 anti-mouse CD452 Antibody.pdf&pdfgen=true

CD62L FITC MEL-14 Biolegend 104406 https://www.biolegend.com/en-us/global-elements/pdf-popup/fitc-anti-mouse-cd62l-antibody-384?filename=FITC anti-mouse CD62L Antibody.pdf&pdfgen=true

CD62L PE MEL-14 Biolegend 104408 https://www.biolegend.com/en-us/products/pe-anti-mouse-cd62l-antibody-386?pdf=true&displayInline=true&leftRightMargin=15&topBottomMargin=15&filename=PE%20anti-mouse%20CD62L%20Antibody.pdf

CD69 PE-Cy7 H1.2F3 Biolegend 104512 https://www.biolegend.com/en-us/global-elements/pdf-popup/pe-cy7-anti-mouse-cd69-antibody-3168?filename=PECy7 anti-mouse CD69 Antibody.pdf&pdfgen=true

CD69 BV650 H1.2F3 Biolegend 104541 https://www.biolegend.com/en-us/products/brilliant-violet-650-anti-mouse-cd69-antibody-13310?pdf=true&displayInline=true&leftRightMargin=15&topBottomMargin=15&filename=Brilliant%20Violet%20650™%20anti-mouse%20CD69%20Antibody.pdf

CD8a SB600 53-6.7 eBioscience 63-0691-82 https://www.thermofisher.com/order/genome-database/dataSheetPdf?producttype=antibody&productsubtype=antibody_primary&productId=63-0691-82&version=102

CD8a APC 53-6.7 Biolegend 100712 https://www.biolegend.com/en-us/products/apc-anti-mouse-cd8a-antibody-150?pdf=true&displayInline=true&leftRightMargin=15&topBottomMargin=15&filename=APC%20anti-mouse%20CD8a%20Antibody.pdf

CD8a BV711 53-6.7 Biolegend 100748 https://d1spbj2x7qk4bg.cloudfront.net/en-ie/products/brilliant-violet-711-anti-mouse-cd8a-antibody-7926?pdf=true&displayInline=true&leftRightMargin=15&topBottomMargin=15&filename=Brilliant%20Violet%20711™%20anti-mouse%20CD8a%20Antibody.pdf&v=20230630102944

Eomes AF488 DAN11MAG eBioscience 53-4875-80 https://www.thermofisher.com/order/genome-database/dataSheetPdf?producttype=antibody&productsubtype=antibody_primary&productId=53-4875-80&version=102

FoxP3 AF488 MF-14 Biolegend 126406 https://www.biolegend.com/en-us/products/alexa-fluor-488-anti-mouse-foxp3-antibody-4661?pdf=true&displayInline=true&leftRightMargin=15&topBottomMargin=15&filename=Alexa%20Fluor®%20488%20anti-mouse%20FOXP3%20Antibody.pdf

KLRG1 (MAFA) BV421 2F1/KLRG1 Biolegend 138414 https://www.biolegend.com/en-us/global-elements/pdf-popup/brilliant-violet-421-anti-mouse-human-klrg1-mafa-antibody-7528?filename=Brilliant Violet 421trade anti-mousehuman KLRG1 MAFA Antibody.pdf&pdfgen=true

ST2 PECy7 DIH9 Biolegend 146610 https://www.biolegend.com/en-us/products/pe-cyanine7-anti-mouse-il-33ralpha-st2-antibody-15505?pdf=true&displayInline=true&leftRightMargin=15&topBottomMargin=15&filename=PE/Cyanine7%20anti-mouse%20IL-33Rα%20(ST2)%20Antibody.pdf

T-bet PECy7 4B10 Biolegend 644824 https://www.biolegend.com/en-us/global-elements/pdf-popup/pe-cy7-anti-t-bet-antibody-8328?filename=PECy7 anti-T-bet Antibody.pdf&pdfgen=true

GATA-3 PE TWAJ eBioscience 14-9966-82 https://www.thermofisher.com/order/genome-database/dataSheetPdf?producttype=antibody&productsubtype=antibody_primary&productId=14-9966-82&version=237

RORgt PE B2D eBioscience 12-6981-82 https://www.thermofisher.com/order/genome-database/dataSheetPdf?producttype=antibody&productsubtype=antibody_primary&productId=12-6981-82&version=237

RORgt APC B2D eBioscience 17-6981-82 https://www.thermofisher.com/order/genome-database/dataSheetPdf?producttype=antibody&productsubtype=antibody_primary&productId=17-6981-82&version=237

IFNg APC XMG1.2 Biolegend 505810 https://www.biolegend.com/it-it/products/apc-anti-mouse-ifn-gamma-antibody-993?pdf=true&displayInline=true&leftRightMargin=15&topBottomMargin=15&filename=APC%20anti-mouse%20IFN-γ%20Antibody.pdf&v=20220831123135

IFNg FITC XMG1.2 Biolegend 505806 https://www.biolegend.com/nl-nl/products/fitc-anti-mouse-ifn-gamma-antibody-995?pdf=true&displayInline=true&leftRightMargin=15&topBottomMargin=15&filename=FITC%20anti-mouse%20IFN-γ%20Antibody.pdf&v=20220831123135

IL-4 PE 11B11 Biolegend 504104 https://www.biolegend.com/de-de/products/pe-anti-mouse-il-4-antibody-893?pdf=true&displayInline=true&leftRightMargin=15&topBottomMargin=15&filename=PE%20anti-mouse%20IL-4%20Antibody.pdf&v=20220913063022

IL-13 PE Cy7 eBio13A eBioscience 25-7133-82 https://www.thermofisher.com/order/genome-database/dataSheetPdf?producttype=antibody&productsubtype=antibody_primary&productId=25-7133-82&version=251

IL-17A PerCPCy5.5 TC11-18H10.1 Biolegend 506920 https://www.biolegend.com/ja-jp/products/percp-cyanine5-5-anti-mouse-il-17a-antibody-4439?pdf=true&displayInline=true&leftRightMargin=15&topBottomMargin=15&filename=PerCP/Cyanine5.5%20anti-mouse%20IL-17A%20Antibody.pdf&v=20220606093917

TCRb PacificB H57-597 Biolegend 109226 https://www.biolegend.com/en-us/global-elements/pdf-popup/pacific-blue-anti-mouse-tcr-beta-chain-antibody-4538?filename=Pacific Bluetrade anti-mouse TCR beta chain Antibody.pdf&pdfgen=true

	TCRb PerCP/Cy5.5 H57-597 Biolegend 109228 https://www.biolegend.com/en-us/global-elements/pdf-popup/percpcyanine55-anti-mouse-tcr-beta-chain-antibody-5603?filename=PerCPCyanine55 anti-mouse TCR beta chain Antibody.pdf&pdfgen=true<br><br>TCRb BV785 H57-597 Biolegend 109249 https://www.biolegend.com/en-us/products/brilliant-violet-785-anti-mouse-tcr-b-chain-antibody-17614?pdf=true&displayInline=true&leftRightMargin=15&topBottomMargin=15&filename=Brilliant%20Violet%20785™%20anti-mouse%20TCR%20β%20chain%20Antibody.pdf
Validation	Validation was provided by the Supplier, all antibodies used are readily available and have been thoroughly tested by the field. Links to TDS has been provided. Please follow the link or go the the manufactures web-page to find all relevant information, testing and references.

Animals and other research organisms

Policy information about studies involving animals; ARRIVE guidelines recommended for reporting animal research, and Sex and Gender in Research

Laboratory animals	All studies involved male and female mice of the C57Bl/6J strain, between the ages of 12-25 weeks. Lines used are: C57BL6/J and C57BL6/J CD45.1 mice were purchased from Charles River, France. Tbx21f/f (Tbx21tm2Srnr) were kindly provided by Dr Steven L Reiner, RORgtf/f (Jackson Laboratories), Foxp3eYFP-Cre (Foxp3tm4(YFP/icre)Ayr) was kindly provided by Dr Alexander Y Rudensky, Rosa26-tdRFP was kindly provided by Dr Hans Jorg Fehling. Foxp3-Cre TGFb f/f mice bone marrow from 6 week-old mice were kindly provided by Dr. Julien Marie. The methods section contains references to the original publications of mice used. Stage 3 Nippostrongylus brasiliensis larvae were kindly provided by Dr Judith E Allen.
Wild animals	No wild animals were used.
Reporting on sex	Both male and female mice were used with no specific bias according to the age threshold and genotype defined.
Field-collected samples	The study did not involve field-collected samples.
Ethics oversight	All animal experimentation complied with regulations of the Direção-Geral de Alimentação e Veterinária (DGAV) Portugal and local ethical review committee and guidelines (iMM-ORBEA).

Note that full information on the approval of the study protocol must also be provided in the manuscript.

Flow Cytometry

Plots

Confirm that:

- [x] The axis labels state the marker and fluorochrome used (e.g. CD4-FITC).
- [x] The axis scales are clearly visible. Include numbers along axes only for bottom left plot of group (a 'group' is an analysis of identical markers).
- [x] All plots are contour plots with outliers or pseudocolor plots.
- [x] A numerical value for number of cells or percentage (with statistics) is provided.

Methodology

Sample preparation	Sample preparation is detailed in methods
Instrument	BD Fortessa X20
Software	Diva and FlowJo
Cell population abundance	N/A
Gating strategy	A gate was first applied on lymphocytes by gating on FSC/SSC, live cells were selected by use of fixable dye (NearIR). Within the live lymphocyte population, required subset of T cells was identified by CD4 or CD8 stain. Treg were identified using Foxp3-Cre-eYFP. Trm cells were identified within the CD8+ population, co-staining for CD69+KLRG1-, and within this CD103+ expression was assessed. Markers used are described in the figure axis.

- [x] Tick this box to confirm that a figure exemplifying the gating strategy is provided in the Supplementary Information.
